# Dental pulp stem cells and Bonelike^®^ for bone regeneration in ovine model

**DOI:** 10.1093/rb/rby025

**Published:** 2018-12-22

**Authors:** J M Campos, A C Sousa, A R Caseiro, S S Pedrosa, P O Pinto, M V Branquinho, I Amorim, J D Santos, T Pereira, C M Mendonça, A Afonso, L M Atayde, A C Maurício

**Affiliations:** 1 Departamento de Clínicas Veterinárias, Instituto de Ciências Biomédicas de Abel Salazar (ICBAS), Universidade do Porto (UP), Rua de Jorge Viterbo Ferreira, no 228, Porto, Portugal; 2 Centro de Estudos de Ciência Animal (CECA), Instituto de Ciências, Tecnologias e Agroambiente da Universidade do Porto (ICETA), Rua D. Manuel II, Apartado 55142, Porto, Portugal; 3 Escola Universitária Vasco da Gama (EUVG), Hospital Veterinário Universitário de Coimbra (HVUC), Campo Universitário – Bloco B, Lordemão, Coimbra, Portugal; 4 REQUIMTE/LAQV – U. Porto – Porto/Portugal, Departamento de Engenharia Metalúrgica e Materiais, Faculdade de Engenharia, Universidade do Porto, Rua, Dr. Roberto Frias, s/n, Porto, Portugal; 5 Faculdade de Engenharia da Universidade do Porto (FEUP), Rua Dr. Roberto Frias, Porto, Portugal; 6 Department of Pathology and Molecular Immunology of the Institute of Biomedical Sciences Abel Salazar (ICBAS), University of Porto, Porto, Portugal; 7 Institute for Research and Innovation in Health, (i3S), University of Porto, Porto, Portugal; 8 Institute of Molecular Pathology and Immunology of the University of Porto (IPATIMUP), Porto, Portugal; 9 Faculdade de Medicina Dentária da Universidade do Porto (FMDUP), Porto, Portugal

**Keywords:** biomaterial, Bonelike^®^, bone regeneration, dental pulp, human bone, hydroxyapatite, mesenchymal stem cells, ovine model, tissue regeneration, tricalcium phosphate

## Abstract

Development of synthetic bone substitutes has arisen as a major research interest in the need to find an alternative to autologous bone grafts. Using an ovine model, the present pre-clinical study presents a synthetic bone graft (Bonelike^®^) in combination with a cellular system as an alternative for the regeneration of non-critical defects. The association of biomaterials and cell-based therapies is a promising strategy for bone tissue engineering. Mesenchymal stem cells (MSCs) from human dental pulp have demonstrated both *in vitro* and *in vivo* to interact with diverse biomaterial systems and promote mineral deposition, aiming at the reconstruction of osseous defects. Moreover, these cells can be found and isolated from many species. Non-critical bone defects were treated with Bonelike^®^ with or without MSCs obtained from the human dental pulp. Results showed that Bonelike^®^ and MSCs treated defects showed improved bone regeneration compared with the defects treated with Bonelike^®^ alone. Also, it was observed that the biomaterial matrix was reabsorbed and gradually replaced by new bone during the healing process. We therefore propose this combination as an efficient binomial strategy that promotes bone growth and vascularization in non-critical bone defects.

## Introduction

In recent years, the average life expectancy of the European population has increased [1] and so has the number of degenerative diseases, osteogenic disorders and bone fractures [2]. More than two million bone grafts were performed throughout the world, making bone grafts the second most performed tissue transplant after blood, in the last decades [3, 4]. According to their origin, bone grafts can be classified as autografts (originating from the individual), allografts (from another individual) and xenografts (from another species) [5]. These different types of bone grafts may present some drawbacks such as limited quantity and availability, donor morbidity and disease transmission. Therefore, alternatives are necessary to overcome these limitations. Synthetic bone substitutes, mainly based on hydroxyapatite (HA) and tricalcium phosphate (TCP), are a good alternative and have been the target of much research and development in recent years. These substitutes replicate the biological properties of natural bone without the disadvantages stated above. Natural bone morphology and properties are usually used as a model in the development of the ideal bone replacement [6], especially with regards to osteoconductive, osteogenesis and osteoinductive properties [3, 7]. The main application of synthetic bone substitutes is to become an alternative in the tackling of critical and non-critical bone defects [8]. Santos and collaborators [9–11] developed a synthetic bone substitute that has been patented and it’s commercially available as Bonelike^®^ (Biosckin S.A., Portugal) [8–11]. Bonelike^®^ is composed of modified calcium phosphate and controlled proportions of HA, TCP and ionic species, to mimic the chemical and structural composition of human bone [12]. Bonelike^®^ presents enhanced bone adhesion, cellular proliferation, and differentiation properties, and is reabsorbed gradually in a controlled way, as in natural bone remodelling [9–11]. Atayde et al. [6] recently demonstrated several advantages of Bonelike^®^, including easy extrusion through a syringe, and improved osteointegration, osteoconduction and degradation due to the presence of larger pores and a spherical format, which can adapt to bone growth.

The use of osteoconductive biomaterials with mesenchymal stem cells (MSCs) allows the proliferative and differentiation capacities of the latter to work in synergy with scaffolding properties of biomaterials. Human MSCs (hMSCs) characteristics have been defined by the Mesenchymal and Tissue Stem Cell Committee of the International Society for Cellular Therapy (ISCT) [13] as being plastic-adherent when maintained in standard culture conditions, expressing cluster of differentiation (CD) 105, CD73 and CD90, and lacking expression of CD45, CD34, CD14 or CD11b, CD79α or CD19 and human leucocyte antigen (HLA)-DR surface molecules. The lack of expression of HLA-DR surface molecules allows their application in other species without the risk of rejection by the receptor and the need of immunosuppression. Human dental pulp stem cells (hDPSCs) are isolated from dental pulp tissue and are a good source MSCs due to their accessibility, availability throughout life, high proliferation and differentiation capabilities, especially towards the osteogenic lineage and promotion of mineral deposition both *in vitro* and *in vivo*. Furthermore, hDPSCs are capable of secreting bioactive factors (growth factors and cytokines) that modulate the activity of native cells and inflammatory populations [14, 15].

In the present study, hDPSCs were characterized and expanded *in vitro* and concurrently implanted *in vivo* with Bonelike^®^, in standardized non-critical bone defects, for pre-clinical trials using an ovine model. The ovine model was selected because of its phylogenetic proximity to humans in terms of musculoskeletal size and mechanical characteristics. The presence of *Havers* channels and the process of cortical remodelling of bone structure, *plus* the docile temperament of this species, are further advantages. Other important considerations are the low acquisition and maintenance costs of sheep, and the ethical and social approval of their use compared with other mammals [16]. The aim was to monitor and evaluate bone regeneration and remodelling of Bonelike^®^*plus* hDPSCs over time in non-critical defects, including the examination of biomaterial reabsorption, bone growth and structure of regenerated areas using histological processing and histomorphometric analysis.

## Materials and methods

### Bonelike^®^ preparation

The Bonelike^®^ were prepared as detailed in [6]. Briefly: HA and P_2_O_5_-CaO based glass were individually prepared and mixed. HA was prepared through a precipitation method consisting of the reaction between calcium hydroxide (Ca(OH)_2_) and ortho-phosphoric acid (H_3_(PO_4_)_2_). A P_2_O_5_–CaO based glass with the composition of 65P_2_O_5_- 5CaO-10CaF_2_-10Na_2_O in mol% was prepared from reagent grade chemicals by using a platinum crucible heated at 1450°C. Bonelike^®^ was obtained by adding 2.5 wt% of glass HA and mixed with a pore forming agent. The mixture was extruded and spheronized and the pellets sintered. Standard sieving techniques were used to obtain the 250–500 μm particle size ranges.

To characterize the Bonelike^®^, a scanning electron microscopy (SEM) was used. The SEM exam was performed using a High-resolution (Schottky) Environmental SEM with X-ray microanalysis and electron backscattered diffraction analysis: Quanta 400 FEG ESEM/EDAX Genesis X4M^®^.

### hMSC preparation

#### Cell culture and maintenance

hDPSCs were obtained from AllCells, LLC (Cat. DP0037F, Lot No. DPSC090411-01). Cells were thawed and expanded *in vitro* using standard protocols previously reported [17–29]. hPDSCs were maintained in αMEM, with GlutaMAX™, without nucleosides (32561029, Gibco^®^) supplemented with 10% (v/v) fetal bovine serum (A31608-02, Gibco^®^), 100 IU/ml penicillin, 0.1 mg/ml streptomycin (15140122, Gibco^®^), 2.05 µg/ml amphotericin B (15290026, Gibco^®^) and 10 mM 4-(2-hydroxyethyl)-1-piperazineethanesulfonic acid Buffer solution (15630122, Gibco^®^), kept at 37°C and in a 95% humidified atmosphere with 5% CO_2_.

#### DPSC characterization

##### DPSC phenotype identity

Surface marker profiles of hDPSCs were assessed by flow cytometry. hDPSCs populations were cultured for 5 days, as described previously [30], harvested using Accutase^TM^ Cell detachment solution (561527, BD Biosciences^©^), being counted and resuspended in Stain Buffer (554676, BD Biosciences^©^). hDPSCs in Passage 5 and 7 (P5 and P7) were incubated with anti-positive (CD90, CD105 and CD44) and anti-negative marker (CD34, CD11b, CD19, CD45 and MHC Class II) antibodies and assayed as per manufacturer’s instructions (hMSC Analysis Kit, 562245, BD Biosciences^©^), using a BD FACSCalibur™ 3 CA Becton Dickinson (BD Biosciences^©^). Flow cytometry data was processed using FlowJo Engine X10.4 (v3.05478, LLC).

##### Gene expression

Reverse transcriptase polymerase chain reaction (RT-PCR) and quantitative PCR (qPCR) were performed for MSCs’ related genes sequences from Bio Rad^®^: CD34 (qHsaCID0007456), CD90/THY1 (qHsaCED0036661), CD73/NT5E (qHsaCID0036556), CD105/ENG (qHsaCID0010800), CD166/ALCAM (qHsaCID0037887), CD117/c-kit (qHsaCID0008692), SOX2 (qHsaCED0036871), OCT3-4/POU5F1 (qHsaCED0038334), MHC Class I/HLA-A (qHsaCED0037388), MHC Class II/HLA-DRA (qHsaCED0037296) and housekeeping genes: β-actin (qHsaCED0036269), glyceraldehyde 3-phosphate dehydrogenase (GAPDH) (qHsaCED0038674).

Cultured hDPSCs in P5–P7 were harvested using Trypsin-EDTA (25200072, Gibco^®^), and pellets of 1 × 10^6^ cells of each group were used for total RNA extraction, using the Aurum^TM^ Total RNA Mini kit (732-6820, Bio Rad^®^) [31]. Briefly, cell pellets were lysed, DNA was removed with DNase I enzyme and obtained RNA was eluted. Total RNA was quantified using nanophotometer readings at 260 and 280 nm (NanoPhotometer^TM^ Pearl, Implen GmbH). Once RNA concentrations were tuned between samples, cDNA was synthesized from the purified RNA using the iScript Reverse Transcription kit (170-8891, Bio Rad^®^) and T100 Terma Cyclar Thermocycler (Bio Rad), as per manufacturer’s instructions. qPCR was performed in a CFX96 Touch^TM^ (BioRad^®^) apparatus using the iTAQ^TM^ SYBR^®^ Green Supermix (172-5120, BioRad^®^) and custom PCR plates encompassing duplicates of targeted human genes and a negative control. Recommended PrimePCR cycling protocol was employed: 95° C for 2 min (activation), 40 cycles comprising 95° C for 5 s (denaturation), −60° C for 30 s (annealing), and 65–95°C (0.5°C increments), 5 s/step (melt curve). The number of cycles for each well was recorded. Data was processed using BioRad CFX^®^ Manager Software 3.1 (Bio Rad Laboratories). Fold differences were calculated using the standard ΔCq method with GAPDH and β-actin as housekeeping genes.

##### Multi-lineage differentiation

hDPSCs (P5-P7) were plated onto 24-well plates (8.000 viable cells/cm^2^) and cultured in standard media until reaching a confluency of 70–80% of culture surface. Cells were transitioned onto specific differentiation media for adipogenesis (StemPro^®^ Adipogenesis Differentiation kit, A10070-01, Gibco^®^) and osteogenesis [standard culture medium supplemented with 5 nM Dexamethasone (D8893 – Sigma Aldrich^®^), 250 µM ascorbic acid-2-phosphate (A4403, Sigma Aldrich^®^) and 10 mM β-glycerphoshate (G9422, Sigma Aldrich^®^)]. Undifferentiated controls were maintained in standard media. Media were refreshed every 3 days, for 21 days. After 14 days, cells under adipogenic differentiation were stained with Oil Red O (ORO), for lipid droplet detection. ORO stain was solubilized using 100% isopropanol (59300, Merck Milipore^®^), and its absorbance quantified at 570 nm.

Von Kossa staining was employed to visualize mineral deposition. Cells were fixated and dehydrated with sequentially increasing ethanol concentrations and let dry. Cells were rehydrated and incubated with 2% Silver nitrate solution (85193, Sigma Aldrich^®^**)**, under Ultra-violet light. Sodium thiosulfate 5% (72049, Sigma Aldrich^®^**)** was added and incubated for 3 min. Wells were rinsed and photographic record obtained. Alizarin Red S (ARS) assay was used to determine osteogenic differentiation of hDPSCs, as previously described in [31]. After fixation with 4% formaldehyde, cells were stained with 40 mM ARS (2003999, Milipore^®^). Following a 30-min incubation, unbound dye was removed and ARS was extracted for quantification, with 10% acetic acid (ARK2183, Sigma-Aldrich^®^). Individual absorbance values were measured at 405 nm through the interpolation of standard curve of ARS concentration (µM).

For chondrogenic differentiation, hDPSCs were plated at 2 × 10^4^ viable cells/well onto a 96-well plate. After 48 h, cells were transferred to chondrogenic culture medium (StemPro^®^ Chondrogenesis Differentiation kit, A10071-01, Gibco^®^) and control wells remained in standard supplementation. Media were renewed every other day for 14 days. After 14 days, Proteoglycan’s synthesis by differentiated chondrocytes was assessed though Alcian Blue staining. Cells were fixated with 4% formaldehyde (252931.1315, Panreac^®^ AppliChem), and stained with Alcian Blue (A9186, Sigma^®^) solution. Alcian Blue was removed and cells were rinsed with acetic acid at 3% (v/v)(A6289, Sigma Aldrich^®^). Acidity was neutralized with distilled water. Differentiated cells were observed under inverted microscope. Sulphated glycosaminoglycan (GAGs) production was determined to assess chondrogenic matrix production, following manufacturers’ instructions. GAGs were extracted using Papain (P3125, Sigma-Aldrich^®^), and Blyscan^TM^ dye reagent (Glycosaminoglycan Assay Blyscan™, Biocolor) was used to induce the precipitation of extracted compounds. Precipitate was solubilized and absorbance at 656 nm was recorded, along with a standard curve for the interpolation of GAG concentration (μg/ml).

### *In vivo* bone regeneration assessment

All the animal testing procedures were in conformity with the Directive 2010/63/EU of the European Parliament and the Portuguese DL 113/2013. All the procedures were approved by the ICBAS-UP Animal Welfare Organism of the Ethics Committee and by the Veterinary Authorities of Portugal. Humane end points were followed in accordance to the OECD Guidance Document on the Recognition, Assessment and Use of Clinical Signs as Humane Endpoints for Experimental Animals Used in Safety Evaluation (2000), and adequate measures were taken to minimize pain and discomfort considering human endpoints for animal suffering and distress. The interaction of Bonelike^®^ in bone tissue was evaluated in 12 healthy skeletally-mature Merino sheep (*Ovis aries*) with an average weight of ∼50 kg and aged between 7 and 8 years.

#### Surgical procedure

##### Pre-surgical procedure

Multimodal anaesthesia was employed, and premedication was administered intramuscularly into the lumbar muscles using xylazine (Rompun^®^ 20 mg/ml, Bayer, 0.1 mg/kg), together with butorphanol (Dolorex^®^ 10 mg/ml, MSD, 0.05 mg/kg). Initial sedation allowed trichotomy of the hind limb. Initial antisepsis of the surgical site was performed with 4% iodopovidone (Betadine^®^) scrub solution. Intraoperative intravenous fluid therapy (NaCl 0.9% B Braun^®^) at a maintenance rate was provided. Induction of general anaesthesia was performed by intravenous bolus administration of tiletamine-zolazepam (Zoletil^®^100, Virbac, 5.5 mg/kg). Loco-regional anaesthesia was provided using lidocaine hydrochloride (Anestesin^®^ 2%, Medinfar-Sorológico) administered via an epidural spinal catheter. Final antiseptic preparation of the operative field was performed with the animal in lateral recumbency using 70% ethanol (Aga) and 10% iodopovidone topical solution. Anaesthetic monitoring of cardiorespiratory parameters was performed and recorded, while intravenous top-up bolus of tiletamine-zolazepam was administrated intraoperatively whenever required.

##### Surgical technique

An ∼15 cm skin incision was made lateral to the diaphysis of the femur, from proximal to distal, and the lateral diaphysis of the femur was exposed. In the lateral diaphysis, a row of five holes with a non-critical size defect of 5.0-mm diameter was drilled through the cortex and into the medulla using a micro-burr (HG.28 Handpiece coupled with an M-SR-FCT motor, Foredom^®^). Continuous flushing with 0.9% sterile saline solution was performed during drilling to minimize thermal damage and to remove any residual bone fragments. A minimal distance of 1 cm was kept between drilled holes to reduce the risk of iatrogenic fracture, and to allow multiple serial analyses of the biomaterial in the same *in vivo* conditions.

##### Biomaterial and cellular system application

The bone defects were assigned to either untreated (control), biomaterial (Bonelike^®^) associated with Tisseel Lyo^®^(Baxter), or Bonelike^®^*plus* Tisseel Lyo^®^ and hDPSCs study groups. Bonelike^®^ granules were combined with Tisseel Lyo^®^ to help retaining the biomaterial and hDPSCs within the defect, working as a cellular-based therapy vehicle. Tisseel Lyo^®^ is a fibrin glue of human origin, comprised of fibrinogen and thrombin and the resultant fibrin clot is very similar to that of normal blood coagulation. It is completely biodegraded by the host’s normal anti-fibrinolytic pathways and does not induce any systemic or local rejection reaction. In the cell-treated defects, hDPSCs (obtained by *in vitro* culture, as described above) were homogenously incorporated in the Bonelike^®^ and Tisseel Lyo^®^ mixture at a cell density of 10 × 10^4^ cells per defect.

##### Post-surgical procedures

Post-operative radiographic follow-up was performed to assess and monitor the surgical defects. After recovery from anaesthesia, animals were transferred to an individual feeding cage for 24 h in order to minimize movements immediately after the surgery. Afterwards they were transferred to a straw yard, with two or three other animals. The intramuscular antibiotic Pendistrep^®^ (6000 IU/kg of benzylpenicillin procaine and 7.5 mg/kg of dihydrostreptomycin, Syva^®^) and a non-steroidal anti-inflammatory drug (Carprofen-Rimadyl^®^, 50 mg/ml, Zoetis, 0.7mg/kg) were given post-operatively and daily for 5 days. To assess for *in loco* bone regeneration phenomena, animals were sacrificed at different time points as described below. This was accomplished in a quiet and isolated environment, under humane conditions using an overdose of intravenous 20% sodium pentobarbital (Eutasil^®^, 200 mg/ml, Ceva). The time-points for the healing process analysis were 30, 60 and 120 days post-operatively, using four animals for each experimental group. Short periods (30 and 60 days) were chosen to observe the initial phase of bone healing, and the longer period (120 days) was performed to allow analysis of the more advanced stages of bone healing, as well as the absorption and degradation of the biomaterial. *Post-mortem*, femurs were dissected and soft tissue was removed from the bone. Radiographic imaging of the collected bones was immediately performed and samples were preserved in 4% formaldehyde. The femur was sectioned to detach each of the five defect sites separately and the samples were subsequently evaluated by histomorphometric and histological analysis.

### Histological processing and histomorphometric analysis

Bone segments were immersed in increasing percentage alcohol solutions (from 70 to 99%) and finally embedded in a Methyl Methacrylate resin (Merck KGaA, Germany). Using a microtome (Accutom, Struers, Denmark) with a diamond blade (TAAB, Griding Wheel Institute, OH, USA), sections were cut in transverse to 150-µm wide and hand-ground to ∼70–80 µm. Sections were then stained with Solochrome Cyanine R (Merk) for histological examination using light microscopy (Eclipse E600, Nikon, Tokyo, Japan), equipped with a calibrated digital camera (Nikon DS-5M-L1 Digital Sight Camera System, Nikon). Unstained slides were used to perform SEM analysis. The SEM and energy dispersive x-ray spectroscopy (EDS) analysis were performed using a high-resolution (Schottky) environmental SEM with X-ray microanalysis and electron backscattered diffraction (Quanta 400 FEG ESEM/EDAX Genesis X4M), operating in high vacuum mode at an accelerating voltage of 15 kV.

According to the technique developed by Atayde et al. [32] the obtained images were divided into three fractions, namely: (i) the periosteal callus (zone of new bone formation over the defect area); (ii) the defect area (square area with its vertices intercepting the defect wall); (3) the endosteal callus (zone of new bone formation underneath the defect area) (Supplementary Fig. S1(1)). Histological images were analysed to evaluate the cellular reaction and the quantity of new bone formation.

#### Threshold analysis

The Threshold analysis technique was optimized in Atayde et al. [6, 32], and herein employed to quantitatively monitor and evaluate bone and biomaterial interactions at the determined time points (30, 60 and 120 days). Briefly, grey scale images obtained from unstained sections were uploaded to the Image J^®^ software (https://imagej.nih.gov/ij/), converted to 8-bit and run through the Threshold tool (Image/Adjust/Threshold). Maximum and minimum threshold values were defined to separate and measure fraction areas of the lacunae/unfilled zone, new bone and biomaterial (Supplementary Fig. S1(2))). Finally, pergentage (%) of new bone [(defect new bone area/defect total area*100], Bonelike^®^ [(defect Bonelike^®^ area/defect total area*100], Unfilled area [(defect Unfilled area/defect total area*100], and Lacunae area [(defect Lacunae area/defect total area*100]. Bone growth at different times post-implantation was also assessed in the different fractions (defect area, periosteal callus and endosteal callus).

### Statistical analysis

Statistical analysis was performed using GraphPad Prism^®^ (version 6.00 for Mac OS X, GraphPad Software, La Jolla, CA, USA). Results were presented as mean ± standard error of the mean (SEM). Comparisons between groups were performed by one-way analysis of variance followed by Tukey’s multiple comparisons test. Differences were considered statistically significant when *P* ≤ 0.05. Significant results between groups were presented using the symbol (*). Significance results are also indicated according to *P* values with one, two, three or four of the symbols (*) corresponding to 0.01< *P* ≤0.05, 0.001 < *P* ≤ 0.01, 0.0001 < *P* ≤ 0.001 and *P* ≤ 0.0001, respectively.

## Results

### Bonelike^®^ SEM

**Figure 1:**
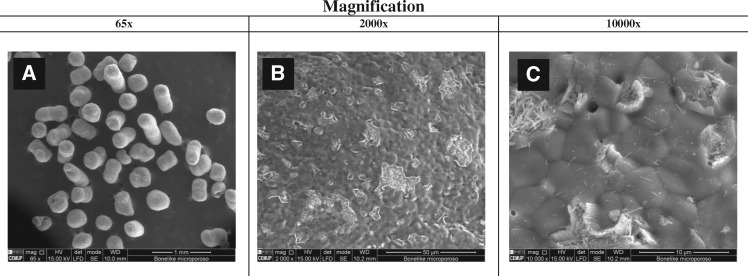
SEM images of Bonelike^®^ granules (Total magnification: **(A)** 65×, **(B)** 2000× and **(C)** 10 000×)

SEM was employed to visualize prepared Bonelike^®^ granules, and detail on its morphology and topography (Fig. 1).

In Fig. 1A the spherical structure of the Bonelike^®^ is observed. At higher magnification, it is possible to visualize a morphology with different roughness levels (Fig. 1B) and the presence of microporosity, homogeneously distributed over the entire sphere (Fig. 1C).

### DPSC characterization

**Figure 2:**
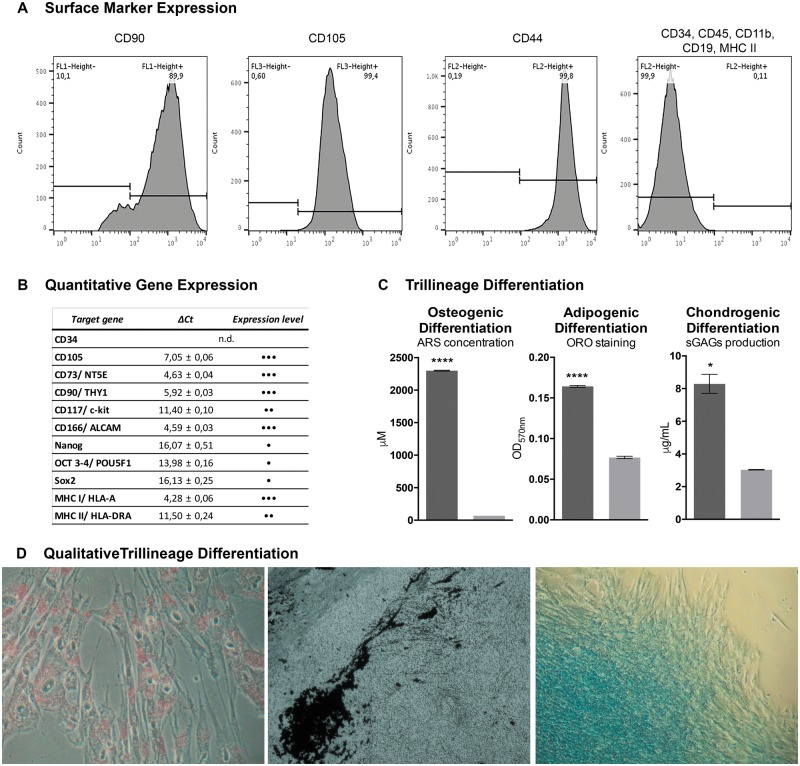
hDPSCs characterization. **(A)** Surface marker expression for hDPSCs identity, assessed by flow cytometry; **(B)** Quantitative gene expression of hMSCs and pluripotency markers, by RT-PCR (•, weak; ••, moderate; •••, strong expression); **(C)** Osteogenic, adipogenic and chondrogenic differentiation determined by ARS, ORO and sulphated GAGs quantification (Significance results are also indicated according to *P* values with one, two, three or four of the symbols (*) corresponding to 0.01< *P* ≤ 0.05, 0.001 < *P* ≤ 0.01, 0.0001 < *P* ≤ 0.001 and *P* ≤ 0.0001, respectively); **(D)** Adipogenic, osteogenic, and chondrogenic differentiation visualized through ORO, von kossa and alcian blue histochemical staining

DPSCs were demonstrated to present characteristic hMSCs’ markers, as assessed through flow cytometry (Fig. 2A**).** Over 90% of the population was positive for CD90, CD105 and CD44, and ≤ 2% were negative for CD34, CD11b, CD19, CD45 and MHC II.

Gene expression was performed through RT-qPCR analysis. Total RNA was successfully extracted from cultured hDPSCs and specific gene expression was assessed (Fig. 2B). CD34 was not detected as expected for hDPSCs. CD105, CD73 and CD90 were highly expressed; CD166, MHC I and CD117 showed strong to moderate expression. Multipotency genes as Nanog, Oct4, Sox2, were also weakly expressed (CT value > 35). Moreover, weak expression of MHC Class II was detected in hDPSCs by RT-qPCR analysis, however, membrane expression demonstrated by flow cytometry, was not detected [33]. Tri-lineage differentiation was quantitatively evaluated through ORO, ARS and GAGs protocols, to evaluate adipogenic, osteogenic and chondrogenic differentiation, respectively. Results demonstrated successful differentiation towards the three lineages, with significant differences from undifferentiated controls (Fig. 2C and D).

### *In vivo* bone regeneration assessment

#### Radiologic study

**Figure 3:**
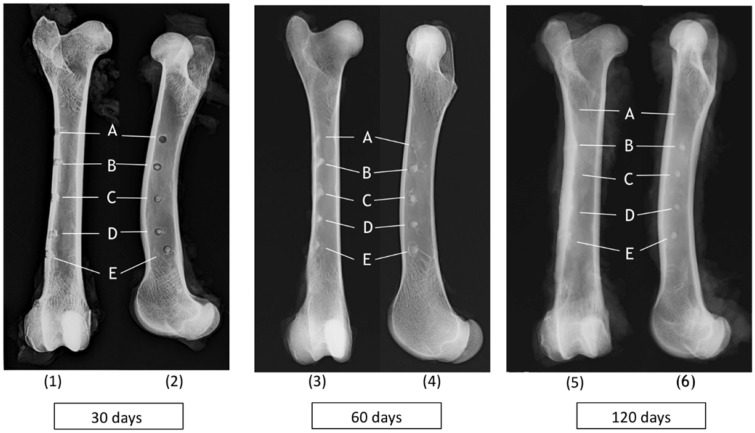
Radiographic images [craniocaudal (1, 3, 5) and mediolateral (2, 4, 6) views] of ovine femurs showing bone regeneration after 30, 60 and 120 days post-implantation of **(A)** untreated control; **(B)** Bonelike^®^; **(C–E)** Bonelike^®^*plus* hDPSCs

Radiographs were obtained in craniocaudal and mediolateral projections of the femur at the different times of implantation (30, 60 and 120 days). Bone defects left untreated (Fig. 3A), treated with Bonelike^®^ (Fig. 3B) and Bonelike^®^*plus* hDPSCs (Fig. 3C–E) are identifiable in Fig. 3.

At the earliest study timepoint (30 days) the defects treated with Bonelike^®^, and Bonelike^®^*plus* hDPSCs, (Fig. 3(1-2), B–E) showed a radiopaque central circle with a radiolucent edge. In the untreated controls, only radiolucent circles were visible (Fig. 3(1-2), A). The radiolucent edge around the defects containing the biomaterial suggests an initial phase of bone regeneration, where osteoclasts remove debris from the bone edges, leading to increased areas of reabsorption [34]. Over time, the radiolucent edges decrease until totally disappearance, representing reabsorption impairment and triggered regeneration (Fig. 3(3-4), A–E). Complete attenuation of the radiolucent edge was seen at 120 days post-implantation, clearly reflecting the transition from the reabsorption phase to the repair phase during the healing process. After 120 days, defects treated with the hDPSCs and Bonelike^®^ (Fig. 3(5-6), C–E), showed significant radiographic evidence of improved bone regeneration, compared with other groups at this healing period.

**Figure 4:**
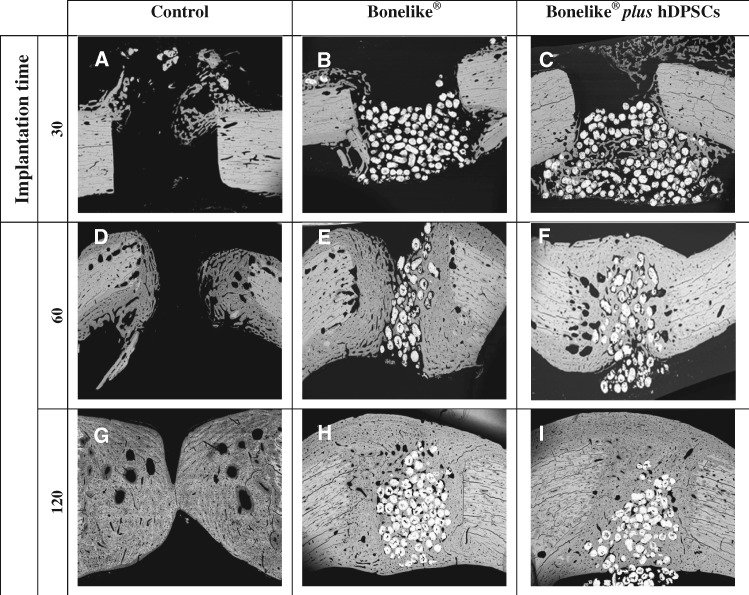
SEM of bone sections representative images from control, Bonelike^®^ and Bonelike^®^*plus* hDPSCs groups at each implantation time (amplification: 30×)

**Figure 5:**
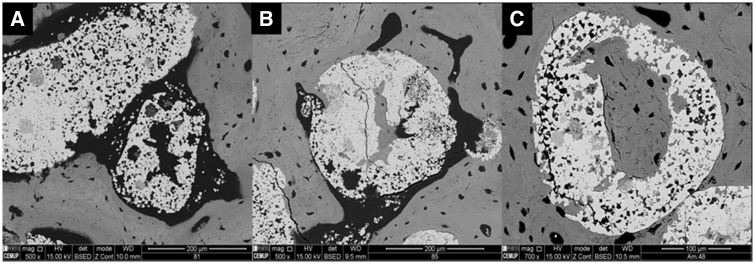
Neo-formed bone inside Bonelike^®^ by SEM analysis of bone defects after 30, 60 and 120 days of implantation. (amplification: **A** and **B**: 500×, **C** 700×). White, —Bonelike^®^; grey, neo-formed bone

**Figure 6:**
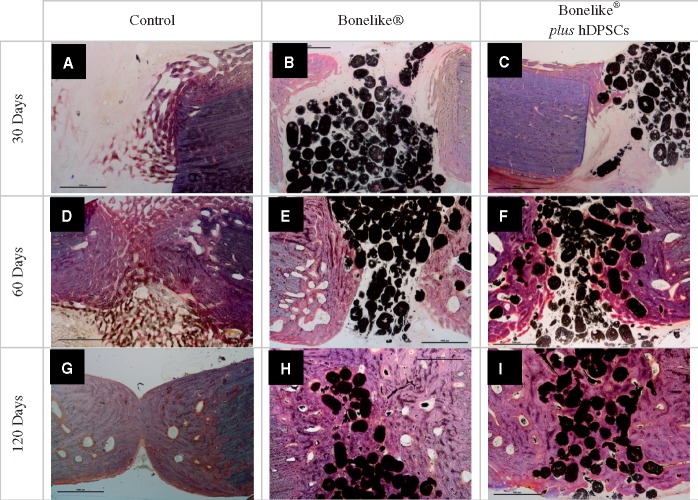
Solochrome cyanine R staining of bone defects sections (control, Bonelike^®^ And Bonelike^®^*plus* hDPSCs), at 30, 60 and 120 days post-implantation. Samples were stained with solochrome cyanine R to differentiate osteoid from newly deposited bone and older bone. Black, Bonelike^®^ spheres; blue, mature bone; pink, neo-formed bone (Amplification 20×)

#### Histological analysis

SEM images were obtained from histological section of the bone samples at 30, 60 and 120 days post-implantation (Fig. 4).

At the earliest time of analysis, the bone defect sites were easily identified, presenting sharp edges in all study groups. In the Bonelike^®^ treated groups, the biomaterial was visualized inside the defect, presenting slight to moderate protrusion into the medullary canal (as evidenced in Fig. 4B and C). The degree of protrusion of Bonelike^®^ into the medullary canal presented great variability between individuals, especially at 30 days post-implantation. At longer time points, this effect was attenuated.

At 60 days post-implantation, the defect edges were softened in all groups, with new tissue formation towards the centre of the defect. In the untreated control and Bonelike^®^ treated groups the newly grown tissue presented an organization distinguishable from the surrounding native bone (Fig. 4D and E, respectively). Contrarily, the groups including hDPSCs within the biomaterial system presented structural features closer to those of the surrounding healthy bone (Fig. 4F). Complete bridging of the opposing edges of the bone defect was only observable in the Bonelike^®^*plus* hDPSCs treated group, at this time period.

At the final 120 days of bone regeneration assessment, the control group samples achieved partial bridging of the defect on the central area of the defect, while the endosteal and periosteal margins remained unbridged (Fig. 4G). Bone resorption *loci* are observable as empty areas within the newly formed tissue. On the other hand, the Bonelike^®^ and Bonelike^®^*plus* hDPSCs treated groups were completely bridged, with the new tissue completely enclosing the bonelike granules. Periosteal and endosteal surfaces continuity was completely restored in both groups (Fig. 4H and I).

Higher magnification SEM imaging enabled the visualization of further details, focussing on the biomaterial–tissue interface. Initially, an increase in porosity was noted in the biomaterial–bone interface, with the formation of voids within the particles (Fig. 5A). With increasing time post-implantation, these voids were replaced by mineralized tissue (Fig. 5B and C). Thus, it was possible to observe the inclusion of Bonelike^®^ in the area of new bone and its presence within newly formed gaps (osteointegration), without evident adverse effects on the surrounding tissue (Fig. 5C). The spherical shape and the granulometry of Bonelike^®^ appeared to be atraumatic to the surrounding hard tissues, and to naturally adapt to the circular pattern of bone growth.

EDS analysis (Supplementary Fig. S2) was performed in order to address the chemical composition of the material within the biomaterial with the newly formed bone, mature bone and Bonelike^®^ itself. EDS analysis revealed a peak of sodium (Na^+^) and magnesium (Mg^2+^) within the biomaterial granules, characteristic of old and newly formed bone.

Histological sections were stained with Solochrome Cyanine R, to allow bone tissue visualization and remodelling assessment.

At 30 days post-implantation, untreated control sections presented very small amounts of newly formed bone, that was predominantly of immature nature (woven and trabecular bone) (Fig. 6A). Contrariwise, in the defects treated with the biomaterial, a higher proportion of compact new bone with a mature lamellar configuration was observed. Furthermore, Haversian systems were observed (Fig. 6B and C). Even as early as 30 days post-implantation, these sections showed good osteointegration, with new bone opposed to the granules and encircling osteocytes. As post-implantation time progressed, bone ingrowth became more evident (Fig. 6D–I); also the formation of Haversian systems and lamellas encircling and encroaching on the Haversian canals are observed.

Bonelike^®^ and Bonelike^®^*plus* hDPSCs-treated defects demonstrated good osteointegration, with the formation of new bone around biomaterial particles, in early implantation times. At 30 days post-implantation, initial absorption events were observed, with a low degree of biomaterial degradation, while at 120 days post-implantation it was possible to observe a high absorption and degradation rate, and biomaterial spheres contour alteration.

### Histomorphometric analysis

Bone sections submitted to histological processing were further analysed histomorphometrically to evaluate the osteo-integration, osteoconduction and degree of absorption on the studied groups, through semi-automatic image segmentation.

**Figure 7:**
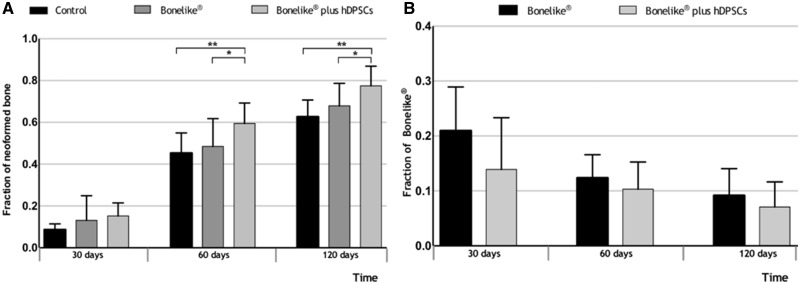
Histomorphometric analysis of biomaterial-bone interface in the defect area. Results presented as mean ± SD of the neo-formed bone **(A)** and Bonelike^®^**(B)** fractions at defect area in the control, Bonelike^®^ and Bonelike^®^*plus* hDPSCs groups, at 30, 60 and 120 days post-implantation (*0.01 < *P* ≤ 0.05; **0.001 < *P* ≤ 0.01)

At all times (30, 60 and 120 days), the results demonstrate that samples treated with the addition of the hDPSCs system depicted increased new bone formation. A greater increase in new bone is observed from 30 to 60 days, when compared with 60–120 days (Fig. 7A) [30 days: untreated control (8.6%), Bonelike^®^ (13.1%) and Bonelike^®^*plus* hDPSCs (15.2%); 60 days: untreated control (45.3%), Bonelike^®^ (48.5%) and Bonelike^®^*plus* hDPSCs (59.4%); 120 days: untreated control (62.6%), Bonelike^®^ (67.9%), and Bonelike^®^*plus* hDPSCs (77.5%)].

The degree of reabsorption of the biomaterial was quantified by comparing the variation of the area occupied by the biomaterial throughout time regarding the different groups (Fig. 7B). Results show that the amount of Bonelike^®^ decreased over time post-implantation, as it was gradually replaced by new bone, confirming its biodegradable properties. Although not statistically different, defects treated with biomaterial alone showed a tendency to maintain higher content of Bonelike^®^ when compared with the hDPSCs inclusive formulation, suggesting decreased biomaterial reabsorption. By analysing the percentage of unfilled area (Supplementary Fig. S3) it is evident that biomaterial-treated defects showed higher percentage of filled area, than controls (Supplementary Tables S1–S3).

Importantly, at 120 days post-implantation, the control sections still had unfilled areas, whereas the treatment groups did not. Indeed, control groups showed a higher fraction of lacunae compared with treatment groups, which indicate increased bone fragility in the first. Supplementary Fig. S4 shows an analysis of new bone formation of the different bone areas (periosteal *callus*, defect area and endosteal *callus*). At 30 and 60 days post-implantation, there is a tendency for a greater production of (immature) bone in biomaterial treated defects (Supplementary Tables S4 and S5). At 120 days post-implantation, there was no variation in bone healing between the three zones (Supplementary Table S6); at this time the bone is deemed completely ossified.

## Discussion

Demographic factors, such as the increase in the average life expectancy of worldwide population, have breached the demand for efficient strategies for bone disease therapy [1, 2]. Although bone tissue transplant (grafting) has been the option of choice for severe fractures therapy [3, 4], it still presents relevant limitations, boosting the need for the alternatives, such as synthetic bone substitutes.

Herein, we complemented the work developed by Atayde et al. [6] that demonstrated the osteointegration, osteoconduction and degradation dynamics of the synthetic bone substitute Bonelike^®^, by adding a cellular component to improve the regenerative system.

hDPSCs were selected due to their accessibility and availability throughout life. Further, this specific population presents a tendency towards the osteogenic lineage and has been demonstrated to endure mineral deposition both *in vitro* and *in vivo* [35]. The hDPSCs system herein employed demonstrated to fully comply with the ISCT’s recommendations for the characterization of MSCs population [13]. The population presented negative to CD34 surface marker. This marker is found expressed in primitive pluripotent stem cells, of both stromal and haematopoietic origin, and is commonly regarded as a haematopoietic marker for stem cells populations. Although most authors confirm the CD34^−^ phenotype, CD34^+^ DPSCs populations have been identified and deemed to retain other MSC characteristics. These were further demonstrated to present enhanced bone formation capacity [36, 37].

Gene expression analysis indicated weak expression of MHC Class II expression, but it did not reflect the effective presence of the molecule in the cellular membrane, as demonstrated by flow cytometry [33]. This weak to absent expression of HLA-DR surface molecules supports the allogenic and xenogenic application of these cells, with reduced risk of immune rejection by the receptor and the need of immunosuppression [38], as observed in this experiment. Also, the proposed system demonstrated great efficiency in osteodifferentiation under *in vitro* specific stimuli. The mineralogenic tendency of hDPSCs is supported by their early expression of putative bone-marker (even in primary undifferentiated isolates), as well as their inherent tendency for spontaneous differentiation [39, 40].

Furthermore, hDPSCs have been demonstrated to secrete an extensive array of bioactive factors (growth factors and cytokines) [30] that modulate the activity of native cells and inflammatory populations at lesion sites and therefore unleash the regenerative response.

To assess for the regenerative potential of the synthetic biomaterial Bonelike^®^ in combination with hDPSCs, a standardized non-critical pre-clinical bone defect model was employed. The ovine model was selected due to its phylogenetic proximity to the human species, regarding its musculoskeletal size and biomechanics. Further, bone tissue of both species presents histological similarities, such as the presence and structure of *Havers* channels and the process of cortical remodelling of bone structure [16].

Bone regeneration and remodelling of untreated and treated defects was monitored over 30, 60 and 120 days. Macroscopic radiographic assessment indicated the presence of the biomaterial at the defect site, surrounded by a radiolucent edge. The radiolucent edge around the defects containing the biomaterial suggest an initial phase of bone regeneration, where osteoclasts remove debris from the bone edges, leading to increased areas of reabsorption [34]. Over time, the radiolucent edges decrease until totally disappearing, representing reabsorption impairment and triggered regeneration. Complete attenuation of the radiolucent edge was seen at 120 days post-implantation, clearly reflecting the transition from the reabsorption phase to the repair phase during the healing process. After 120 days, defects treated with the hDPSCs and Bonelike^®^ showed significant radiographic evidence of improved bone regeneration, compared with other groups at 120 days of healing period.

The signs of bone regeneration were further confirmed microscopically. As has been reported, one of the challenges to granular biomaterial application is its maintenance at the lesion/delivery site. Although we associated a fibrin glue component to the Bonelike^®^ granules, we still observed variable degrees of system dislodgement towards the bone marrow cavity. The biomaterial was verified on histology to be within the defect margins, although the existence of some material in the medullary zone was recorded. Also, we observed some degree of inter-subject variability regarding new bone formation reinforcing the need for careful planning of experimental procedures and adequate number of animals per experimental group.

Accelerated defect bridging in the biomaterial filled defects was observed. The biomaterial posed as both a stimuli and scaffold of newly-formed tissue penetration and is progressively embedded within the newly deposited matrix. The observed features confirm other authors previous findings [6]. Biomaterial-treated defects showed faster bone maturation than controls; with lamellar bone formation and arise of Haversian systems. This phenomenon reflects the osteopromotion associated to the porous nature of the biomaterial, which facilitates cell anchoring and protein absorption and consequently better osteoconduction. Indeed, the microtomography (roughness) of the biomaterial has been demonstrated to be of the utmost importance to hDPSCs adhesion and osteopromotion [41], and that microconcave surfaces favour osteodifferentiation and formation of thick mineralized tissue by seeded cells, due to the resemblance to native bone trabecular spaces [42]. The spherical shape and the granulometry of Bonelike^®^ have been previously identified as to naturally adapt circular pattern of native bone growth [6]. These features also enhance cell migration, proliferation of native and delivered cells, as well as improving vascular invasion, resulting in optimized osteointegration and osteo-reabsorption.

The addition of hDPSCs to the therapeutic system resulted in higher bone growth and accelerated defect bridging, ensuring contact of bone edges from the centre to the endosteal surface of the bone. Immunohistochemical staining also denoted increased mature lamellar bone content in Bonelike^®^ treated defects, with improved histological features recovery. The improvement and acceleration of bone regeneration events is particularly evident at the earliest times of regeneration.

Biomaterial reabsorption is neglectable at the first observation time but becomes evident after 4 months. The reabsorption phenomena are indicated by the outline attenuation and bone tissue invasion inside voids created within the biomaterial granules. Reabsorption phenomena occurring mainly by dissolution and enzymatic/cellular activity (osteoclasts), release mineral elements that are re-incorporated in the newly formed bone. The hDPSCs inclusive formulation, tendentially presented inferior Bonelike^®^ content when compared with the cell-free formulation, suggesting increased biomaterial reabsorption rates.

The combination of mineral and non-mineral based biomaterials in association to dental-derived MSC populations aiming at bone regeneration is regarded as an enthusiastic road for therapy development, as revised in [35]. The proposed combinations unanimously provided improved accelerated and increased bone formation, of predominant mature lamellar nature, finally resulting in remodelled bone with near-native histological features [43]. Comparable observations resulted from the presently proposed combination of Bonelike^®^ and hDPSCs.

## Conclusions

This study describes the *in vivo* performance of Bonelike^®^ in association to hDPSCs in an ovine model of non-critical bone defects at 30, 60 and 120 days post-implantation. It demonstrated Bonelike^®^ associated with hDPSCs to be a very promising therapeutic system for bone regeneration. Results obtained by post-mortem bone radiology besides histological and histomorphometric analyses show that defects filled with Bonelike^®^ and hDPSCs had overall superior new bone formation.

The study of this system’s performance in non-critical bone defects is an essential step to its progression towards clinical applications from small defects (such as alveolar defects resulting from dental extractions) up to extensive trauma events (currently addressed through auto-, allo- and xenogeneic bone transplantation). Further studies ought to entail on this system’s performance in increasing defect sizes, aiming at their effective application on clinical scenarios. We envision their inclusion in non-critical fractures treatment, to accelerate and improve regeneration as well enabling the healing of critical and/or non-healing defects.

## Supplementary Material

Supplementary Data
