# Supplementary material for: Dental pulp stem cells and Bonelike® for bone regeneration in ovine model
Source: Regen Biomater. 2018 Dec 22;6(1):49–59. doi: 10.1093/rb/rby025 (PMC6362823; doi:10.1093/rb/rby025)
Supplement: Supplementary Data [file rby025_supp.zip › rby025-suppl_data/rby025_Supplementary_data.docx]

**Supplementary Information**


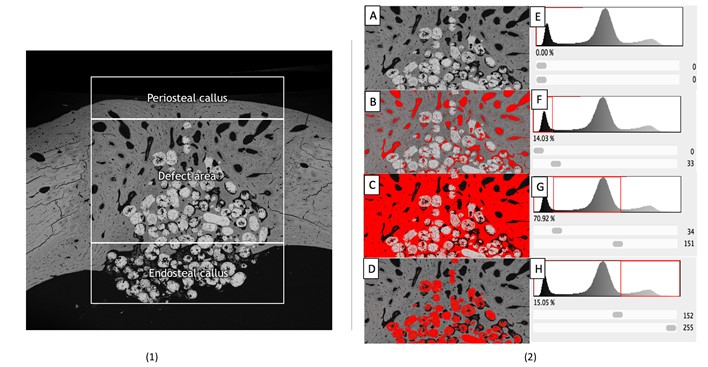


**Figure S1:** SEM image of bone defect divided into three different fractions: periosteal callus, defect area and endosteal callus (Amplification: 30x). Threshold analysis of a SEM image of bone tissue where Bonelike^®^ **(A - D)** was implanted. The image segmentation was performed using histograms (E - H). A – Original image; B – With bone gaps highlighted (red); C – With bone highlighted (red); D – With Biomaterial highlighted (red).


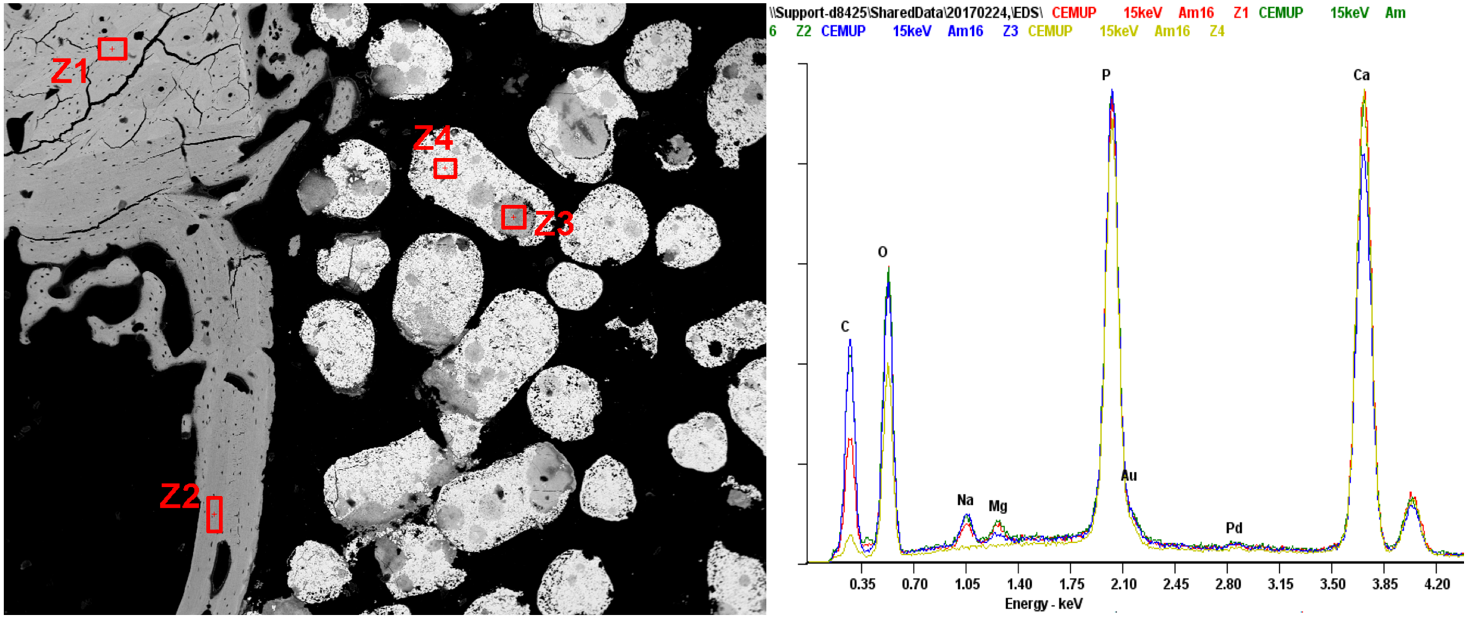


**Figure S2:** Image SEM of bone defect with respective EDS at 30 days of implantation. Red - Old bone (Z1); Dark green – Neo-formed bone (Z2); Blue - material incorporated inside Bonelike® (Z3); Yellow - Bonelike^®^ (Z4) (Amplification: 100x).


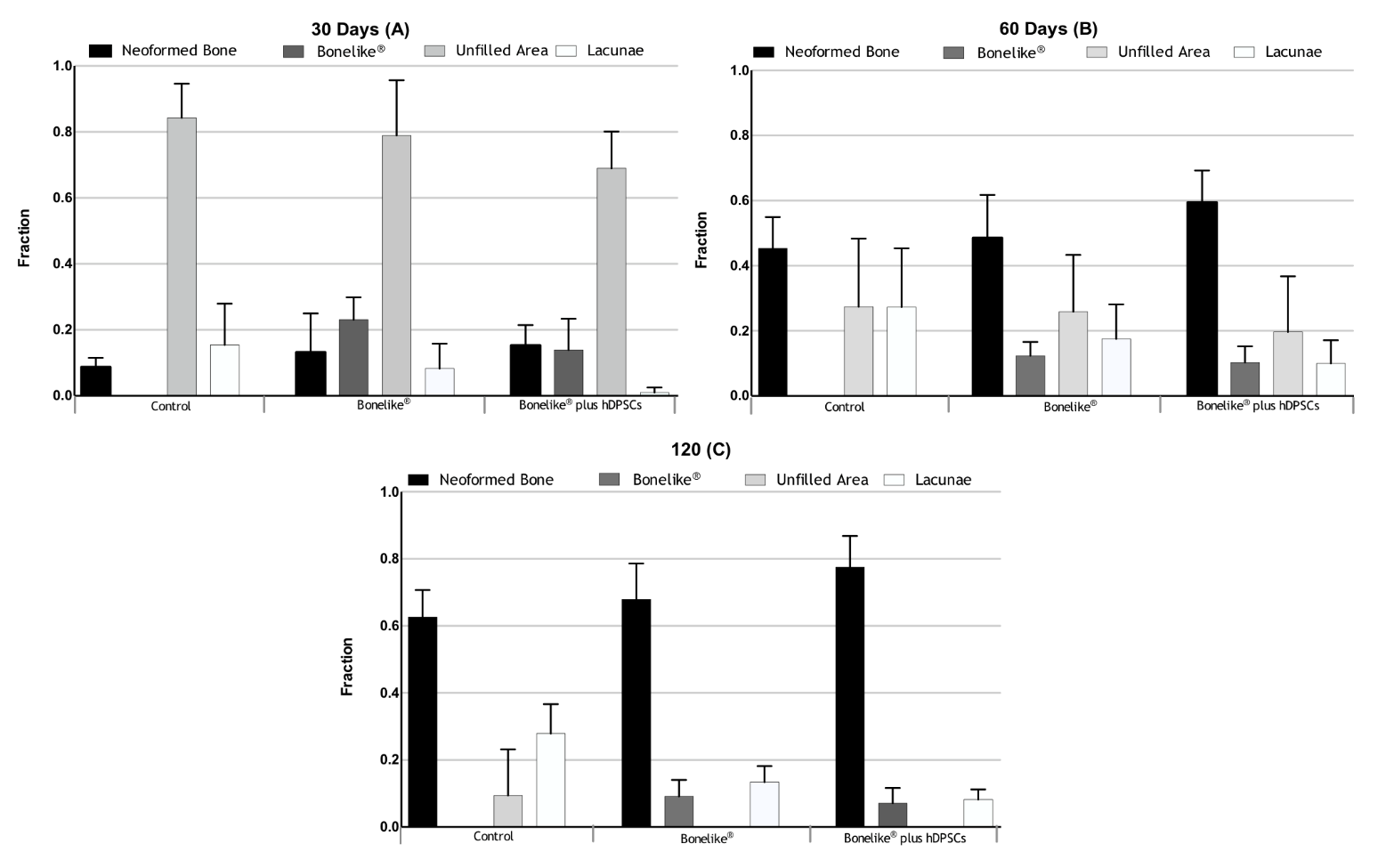


**Figure S3:** Histomorphometric analysis of bone defects with the Mean ± Standard deviation of the neo-formed bone, Bonelike^®^, unfilled area and lacunae fractions, at defect area, in 30 days (graphic A), 60 days (graphic B) and 120 days (graphic C).

**Table S1:** Statistical significance of histomorphometric results at 30 days of implantation. Significance of the results is indicated according to P values with one, two, three or four of the symbols (*) corresponding to 0.01≤P<0.05; 0.001≤P< 0.01; 0.0001≤P<0.001 and P<0.0001, respectively; ns, not significant.

| ***Histomorphometric***  ***Statistical significance*** | | | ***30 days*** | | | | | | | | | | | |
| --- | --- | --- | --- | --- | --- | --- | --- | --- | --- | --- | --- | --- | --- | --- |
|  |  |  | ***Control*** | | | | ***Bonelike®*** | | | | ***Bonelike® plus hDPSCs*** | | | |
|  |  |  | Neoformed Bone | Bonelike® | Unfilled Area | Lacunae | Neoformed Bone | Bonelike® | Unfilled Area | Lacunae | Neoformed Bone | Bonelike® | Unfilled Area | Lacunae |
| ***30 days*** | ***Control*** | Neoformed Bone |  | ns | **** | ns | ns | ns | **** | ns | ns | ns | **** | ns |
|  |  | Bonelike® |  |  | **** | ns | ns | *** | **** | ns | ** | * | **** | ns |
|  |  | Unfilled Area |  |  |  | **** | **** | **** | ns | **** | **** | **** | ** | **** |
|  |  | Lacunae |  |  |  |  | ns | ns | **** | ns | ns | ns | **** | * |
|  | ***Bonelike®*** | Neoformed Bone |  |  |  |  |  | ns | **** | ns | ns | ns | **** | ns |
|  |  | Bonelike® |  |  |  |  |  |  | **** | ns | ns | ns | **** | **** |
|  |  | Unfilled Area |  |  |  |  |  |  |  | **** | **** | **** | ns | **** |
|  |  | Lacunae |  |  |  |  |  |  |  |  | ns | ns | **** | ns |
|  | ***Bonelike® plus hDPSCs*** | Neoformed Bone |  |  |  |  |  |  |  |  |  | ns | **** | **** |
|  |  | Bonelike® |  |  |  |  |  |  |  |  |  |  | **** | **** |
|  |  | Unfilled Area |  |  |  |  |  |  |  |  |  |  |  | **** |
|  |  | Lacunae |  |  |  |  |  |  |  |  |  |  |  |  |

**Table S2:** Statistical significance of histomorphometric results at 60 days of implantation. Significance of the results is indicated according to P values with one, two, three or four of the symbols (*) corresponding to 0.01≤P<0.05; 0.001≤P< 0.01; 0.0001≤P<0.001 and P<0.0001, respectively; ns, not significant.

| ***Histomorphometric***  ***Statistical significance*** | | | ***60 days*** | | | | | | | | | | | |
| --- | --- | --- | --- | --- | --- | --- | --- | --- | --- | --- | --- | --- | --- | --- |
|  |  |  | ***Control*** | | | | ***Bonelike®*** | | | | ***Bonelike® plus hDPSCs*** | | | |
|  |  |  | Neoformed Bone | Bonelike® | Unfilled Area | Lacunae | Neoformed Bone | Bonelike® | Unfilled Area | Lacunae | Neoformed Bone | Bonelike® | Unfilled Area | Lacunae |
| ***60 days*** | ***Control*** | Neoformed Bone |  | **** | ns | ns | ns | **** | * | *** | ns | **** | **** | **** |
|  |  | Bonelike® |  |  | *** | *** | **** | ns | ** | ns | **** | ns | ** | ns |
|  |  | Unfilled Area |  |  |  | ns | * | ns | ns | ns | **** | * | ns | * |
|  |  | Lacunae |  |  |  |  | * | ns | ns | ns | **** | * | ns | * |
|  | ***Bonelike®*** | Neoformed Bone |  |  |  |  |  | **** | ** | **** | ns | **** | **** | **** |
|  |  | Bonelike® |  |  |  |  |  |  | ns | ns | **** | ns | ns | ns |
|  |  | Unfilled Area |  |  |  |  |  |  |  | ns | **** | ns | ns | * |
|  |  | Lacunae |  |  |  |  |  |  |  |  | **** | ns | ns | ns |
|  | ***Bonelike® plus hDPSCs*** | Neoformed Bone |  |  |  |  |  |  |  |  |  | **** | **** | **** |
|  |  | Bonelike® |  |  |  |  |  |  |  |  |  |  | ns | ns |
|  |  | Unfilled Area |  |  |  |  |  |  |  |  |  |  |  | ns |
|  |  | Lacunae |  |  |  |  |  |  |  |  |  |  |  |  |

**Table S3:** Statistical significance of histomorphometric results at 120 days of implantation. Significance of the results is indicated according to P values with one, two, three or four of the symbols (*) corresponding to 0.01≤P<0.05; 0.001≤P< 0.01; 0.0001≤P<0.001 and P<0.0001, respectively; ns, not significant.

| ***Histomorphometric***  ***Statistical significance*** | | | ***120 days*** | | | | | | | | | | | |
| --- | --- | --- | --- | --- | --- | --- | --- | --- | --- | --- | --- | --- | --- | --- |
|  |  |  | ***Control*** | | | | ***Bonelike®*** | | | | ***Bonelike® plus hDPSCs*** | | | |
|  |  |  | Neoformed Bone | Bonelike® | Unfilled Area | Lacunae | Neoformed Bone | Bonelike® | Unfilled Area | Lacunae | Neoformed Bone | Bonelike® | Unfilled Area | Lacunae |
| ***120 days*** | ***Control*** | Neoformed Bone |  | **** | **** | **** | ns | **** | **** | **** | **** | **** | **** | **** |
|  |  | Bonelike® |  |  | ns | **** | **** | ns | ns | ** | **** | ns | ns | ns |
|  |  | Unfilled Area |  |  |  | **** | **** | ns | ns | ns | **** | ns | * | ns |
|  |  | Lacunae |  |  |  |  | **** | **** | **** | *** | **** | **** | **** | **** |
|  | ***Bonelike®*** | Neoformed Bone |  |  |  |  |  | **** | **** | **** | * | **** | **** | **** |
|  |  | Bonelike® |  |  |  |  |  |  | ns | ns | **** | ns | * | ns |
|  |  | Unfilled Area |  |  |  |  |  |  |  | ** | **** | ns | ns | ns |
|  |  | Lacunae |  |  |  |  |  |  |  |  | **** | ns | **** | ns |
|  | ***Bonelike® plus hDPSCs*** | Neoformed Bone |  |  |  |  |  |  |  |  |  | **** | **** | **** |
|  |  | Bonelike® |  |  |  |  |  |  |  |  |  |  | * | ns |
|  |  | Unfilled Area |  |  |  |  |  |  |  |  |  |  |  | ** |
|  |  | Lacunae |  |  |  |  |  |  |  |  |  |  |  |  |

**
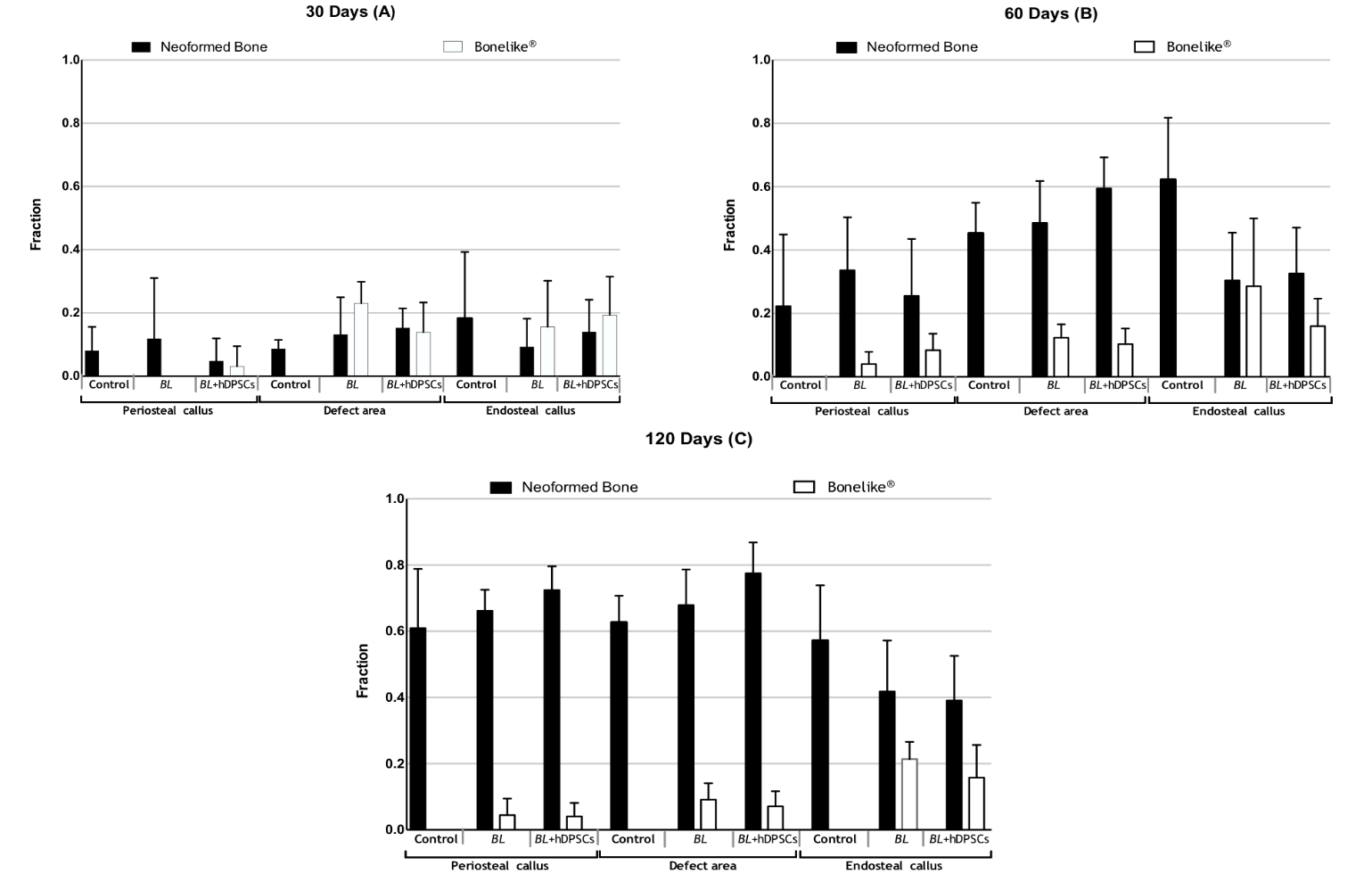
**

**Figure S4:** Histomorphometric analysis of bone defects with the Mean ± Standard deviation of the Neo-formed Bone and Bonelike^®^ with group Control, Bonelike^®^ and Bonelike^®^ plus hDPSCs, divided into three different fractions: periosteal callus, defect area and endosteal callus, in the respective implantation times 30 days (graphic A), 60 days (graphic B) and 120 days (graphic C).

**Table S4:** Statistical significance of histomorphometric results at 30 days of implantation. Significance of the results is indicated according to P values with one, two, three or four of the symbols (*) corresponding to 0.01≤P<0.05; 0.001≤P< 0.01; 0.0001≤P<0.001 and P<0.0001, respectively; ns, not significant.

| ***Histomorphometric***  ***Statistical significance*** | | | | ***30 days*** | | | | | | | | | | | | | | | | | |
| --- | --- | --- | --- | --- | --- | --- | --- | --- | --- | --- | --- | --- | --- | --- | --- | --- | --- | --- | --- | --- | --- |
|  |  |  |  | ***Periosteal callus*** | | | | | | ***Defect area*** | | | | | | ***Endosteal callus*** | | | | | |
|  |  |  |  | ***Control*** | | ***Bonelike®*** | | ***Bonelike® plus hDPSCs*** | | ***Control*** | | ***Bonelike®*** | | ***Bonelike® plus hDPSCs*** | | ***Control*** | | ***Bonelike®*** | | ***Bonelike® plus hDPSCs*** | |
|  |  |  |  | Neoformed Bone | Bonelike® | Neoformed Bone | Bonelike® | Neoformed Bone | Bonelike® | Neoformed Bone | Bonelike® | Neoformed Bone | Bonelike® | Neoformed Bone | Bonelike® | Neoformed Bone | Bonelike® | Neoformed Bone | Bonelike® | Neoformed Bone | Bonelike® |
| ***30 days*** | ***Periosteal callus*** | ***Control*** | Neoformed Bone |  | ns | ns | ns | ns | ns | ns | ns | ns | ns | ns | ns | ns | ns | ns | ns | ns | ns |
|  |  |  | Bonelike® |  |  | ns | ns | ns | ns | ns | ns | ns | ** | * | ns | ns | ns | ns | ns | ns | ** |
|  |  | ***Bonelike®*** | Neoformed Bone |  |  |  | ns | ns | ns | ns | ns | ns | ns | ns | ns | ns | ns | ns | ns | ns | ns |
|  |  |  | Bonelike® |  |  |  |  | ns | ns | ns | ns | ns | * | ns | ns | ns | ns | ns | ns | ns | ** |
|  |  | ***Bonelike® plus hDPSCs*** | Neoformed Bone |  |  |  |  |  | ns | ns | ns | ns | * | * | ns | ns | ns | ns | ns | ns | **** |
|  |  |  | Bonelike® |  |  |  |  |  |  | ns | ns | ns | ** | ** | ** | * | ns | ns | ns | ** | **** |
|  | ***Defect area*** | ***Control*** | Neoformed Bone |  |  |  |  |  |  |  | ns | ns | ns | ns | ns | ns | ns | ns | ns | ns | ns |
|  |  |  | Bonelike® |  |  |  |  |  |  |  |  | ns | ** | ns | ns | ns | ns | ns | ns | * | ** |
|  |  | ***Bonelike®*** | Neoformed Bone |  |  |  |  |  |  |  |  |  | ns | ns | ns | ns | ns | ns | ns | ns | ns |
|  |  |  | Bonelike® |  |  |  |  |  |  |  |  |  |  | ns | ns | ns | ** | ns | ns | ns | ns |
|  |  | ***Bonelike® plus hDPSCs*** | Neoformed Bone |  |  |  |  |  |  |  |  |  |  |  | ns | ns | * | ns | ns | ns | ns |
|  |  |  | Bonelike® |  |  |  |  |  |  |  |  |  |  |  |  | ns | ns | ns | ns | ns | ns |
|  | ***Endosteal callus*** | ***Control*** | Neoformed Bone |  |  |  |  |  |  |  |  |  |  |  |  |  | ns | ns | ns | ns | ns |
|  |  |  | Bonelike® |  |  |  |  |  |  |  |  |  |  |  |  |  |  | ns | ns | ns | ** |
|  |  | ***Bonelike®*** | Neoformed Bone |  |  |  |  |  |  |  |  |  |  |  |  |  |  |  | ns | ns | ns |
|  |  |  | Bonelike® |  |  |  |  |  |  |  |  |  |  |  |  |  |  |  |  | ns | ns |
|  |  | ***Bonelike® plus hDPSCs*** | Neoformed Bone |  |  |  |  |  |  |  |  |  |  |  |  |  |  |  |  |  | ns |
|  |  |  | Bonelike® |  |  |  |  |  |  |  |  |  |  |  |  |  |  |  |  |  |  |

| ***Histomorphometric***  ***Statistical significance*** | | | | ***60 days*** | | | | | | | | | | | | | | | | | |
| --- | --- | --- | --- | --- | --- | --- | --- | --- | --- | --- | --- | --- | --- | --- | --- | --- | --- | --- | --- | --- | --- |
|  |  |  |  | ***Periosteal callus*** | | | | | | ***Defect area*** | | | | | | ***Endosteal callus*** | | | | | |
|  |  |  |  | ***Control*** | | ***Bonelike®*** | | ***Bonelike® plus hDPSCs*** | | ***Control*** | | ***Bonelike®*** | | ***Bonelike® plus hDPSCs*** | | ***Control*** | | ***Bonelike®*** | | ***Bonelike® plus hDPSCs*** | |
|  |  |  |  | Neoformed Bone | Bonelike® | Neoformed Bone | Bonelike® | Neoformed Bone | Bonelike® | Neoformed Bone | Bonelike® | Neoformed Bone | Bonelike® | Neoformed Bone | Bonelike® | Neoformed Bone | Bonelike® | Neoformed Bone | Bonelike® | Neoformed Bone | Bonelike® |
| ***60 days*** | ***Periosteal callus*** | ***Control*** | Neoformed Bone |  | * | ns | ns | ns | ns | * | * | ** | ns | **** | ns | **** | * | ns | ns | ns | ns |
|  |  |  | Bonelike® |  |  | **** | ns | *** | ns | **** | ns | **** | ns | **** | ns | **** | ns | **** | *** | **** | ns |
|  |  | ***Bonelike®*** | Neoformed Bone |  |  |  | **** | ns | **** | ns | **** | ns | * | **** | *** | *** | **** | ns | ns | ns | * |
|  |  |  | Bonelike® |  |  |  |  | ** | ns | **** | ns | **** | ns | **** | ns | **** | ns | ** | ** | **** | ns |
|  |  | ***Bonelike® plus hDPSCs*** | Neoformed Bone |  |  |  |  |  | *** | * | *** | *** | ns | **** | ** | **** | *** | ns | ns | ns | ns |
|  |  |  | Bonelike® |  |  |  |  |  |  | **** | ns | **** | ns | **** | ns | **** | ns | ** | ** | **** | ns |
|  | ***Defect area*** | ***Control*** | Neoformed Bone |  |  |  |  |  |  |  | **** | ns | **** | ns | **** | ns | **** | ns | ns | ns | **** |
|  |  |  | Bonelike® |  |  |  |  |  |  |  |  | **** | ns | **** | ns | **** | ns | **** | *** | **** | ns |
|  |  | ***Bonelike®*** | Neoformed Bone |  |  |  |  |  |  |  |  |  | **** | ns | **** | ns | **** | ns | ns | ns | **** |
|  |  |  | Bonelike® |  |  |  |  |  |  |  |  |  |  | ns | **** | ns | **** | ns | ns | ns | **** |
|  |  | ***Bonelike® plus hDPSCs*** | Neoformed Bone |  |  |  |  |  |  |  |  |  |  |  | **** | ns | **** | **** | **** | **** | **** |
|  |  |  | Bonelike® |  |  |  |  |  |  |  |  |  |  |  |  | **** | ns | ** | * | **** | ns |
|  | ***Endosteal callus*** | ***Control*** | Neoformed Bone |  |  |  |  |  |  |  |  |  |  |  |  |  | **** | **** | **** | **** | **** |
|  |  |  | Bonelike® |  |  |  |  |  |  |  |  |  |  |  |  |  |  | **** | *** | **** | ns |
|  |  | ***Bonelike®*** | Neoformed Bone |  |  |  |  |  |  |  |  |  |  |  |  |  |  |  | ns | ns | ns |
|  |  |  | Bonelike® |  |  |  |  |  |  |  |  |  |  |  |  |  |  |  |  | ns | ns |
|  |  | ***Bonelike® plus hDPSCs*** | Neoformed Bone |  |  |  |  |  |  |  |  |  |  |  |  |  |  |  |  |  | ** |
|  |  |  | Bonelike® |  |  |  |  |  |  |  |  |  |  |  |  |  |  |  |  |  |  |

**Table S5:** Statistical significance of histomorphometric results at 60 days of implantation. Significance of the results is indicated according to P values with one, two, three or four of the symbols (*) corresponding to 0.01≤P<0.05; 0.001≤P< 0.01; 0.0001≤P<0.001 and P<0.0001, respectively; ns, not significant.

| ***Histomorphometric***  ***Statistical significance*** | | | | ***120 days*** | | | | | | | | | | | | | | | | | |
| --- | --- | --- | --- | --- | --- | --- | --- | --- | --- | --- | --- | --- | --- | --- | --- | --- | --- | --- | --- | --- | --- |
|  |  |  |  | ***Periosteal callus*** | | | | | | ***Defect area*** | | | | | | ***Endosteal callus*** | | | | | |
|  |  |  |  | ***Control*** | | ***Bonelike®*** | | ***Bonelike® plus hDPSCs*** | | ***Control*** | | ***Bonelike®*** | | ***Bonelike® plus hDPSCs*** | | ***Control*** | | ***Bonelike®*** | | ***Bonelike® plus hDPSCs*** | |
|  |  |  |  | Neoformed Bone | Bonelike® | Neoformed Bone | Bonelike® | Neoformed Bone | Bonelike® | Neoformed Bone | Bonelike® | Neoformed Bone | Bonelike® | Neoformed Bone | Bonelike® | Neoformed Bone | Bonelike® | Neoformed Bone | Bonelike® | Neoformed Bone | Bonelike® |
| ***120 days*** | ***Periosteal callus*** | ***Control*** | Neoformed Bone |  | **** | ns | **** | ns | **** | ns | **** | ns | **** | ** | **** | ns | **** | ** | **** | **** | **** |
|  |  |  | Bonelike® |  |  | **** | ns | **** | ns | **** | ns | **** | ns | **** | ns | **** | ns | **** | *** | **** | ** |
|  |  | ***Bonelike®*** | Neoformed Bone |  |  |  | **** | ns | **** | ns | **** | ns | **** | ns | **** | **** | **** | **** | **** | **** | ns |
|  |  |  | Bonelike® |  |  |  |  | **** | ns | **** | ns | **** | ns | **** | ns | **** | **** | * | **** | ns | **** |
|  |  | ***Bonelike® plus hDPSCs*** | Neoformed Bone |  |  |  |  |  | **** | ns | **** | ns | **** | ns | **** | ** | **** | **** | **** | **** | **** |
|  |  |  | Bonelike® |  |  |  |  |  |  | **** | ns | **** | ns | **** | ns | **** | ns | **** | *** | **** | ** |
|  | ***Defect area*** | ***Control*** | Neoformed Bone |  |  |  |  |  |  |  | **** | ns | ns | ns | ns | **** | ** | **** | ns | **** | ns |
|  |  |  | Bonelike® |  |  |  |  |  |  |  |  | **** | * | **** | ns | **** | *** | **** | **** | **** | **** |
|  |  | ***Bonelike®*** | Neoformed Bone |  |  |  |  |  |  |  |  |  | ns | **** | ns | **** | ns | **** | *** | **** | ** |
|  |  |  | Bonelike® |  |  |  |  |  |  |  |  |  |  | **** | ns | **** | ns | **** | ns | **** | ns |
|  |  | ***Bonelike® plus hDPSCs*** | Neoformed Bone |  |  |  |  |  |  |  |  |  |  |  | **** | **** | **** | **** | **** | **** | **** |
|  |  |  | Bonelike® |  |  |  |  |  |  |  |  |  |  |  |  | **** | ns | **** | * | **** | ns |
|  | ***Endosteal callus*** | ***Control*** | Neoformed Bone |  |  |  |  |  |  |  |  |  |  |  |  |  | **** | ns | **** | *** | **** |
|  |  |  | Bonelike® |  |  |  |  |  |  |  |  |  |  |  |  |  |  | **** | *** | **** | ** |
|  |  | ***Bonelike®*** | Neoformed Bone |  |  |  |  |  |  |  |  |  |  |  |  |  |  |  | *** | ns | **** |
|  |  |  | Bonelike® |  |  |  |  |  |  |  |  |  |  |  |  |  |  |  |  | *** | ns |
|  |  | ***Bonelike® plus hDPSCs*** | Neoformed Bone |  |  |  |  |  |  |  |  |  |  |  |  |  |  |  |  |  | **** |
|  |  |  | Bonelike® |  |  |  |  |  |  |  |  |  |  |  |  |  |  |  |  |  |  |

**Table S6:** Statistical significance of histomorphometric results at 120 days of implantation. Significance of the results is indicated according to P values with one, two, three or four of the symbols (*) corresponding to 0.01≤P<0.05; 0.001≤P< 0.01; 0.0001≤P<0.001 and P<0.0001, respectively; ns, not significant.
