# Supplementary figures and images for: Dental pulp stem cells and Bonelike® for bone regeneration in ovine model
Source: Regen Biomater. 2018 Dec 22;6(1):49–59. doi: 10.1093/rb/rby025 (PMC6362823; doi:10.1093/rb/rby025)

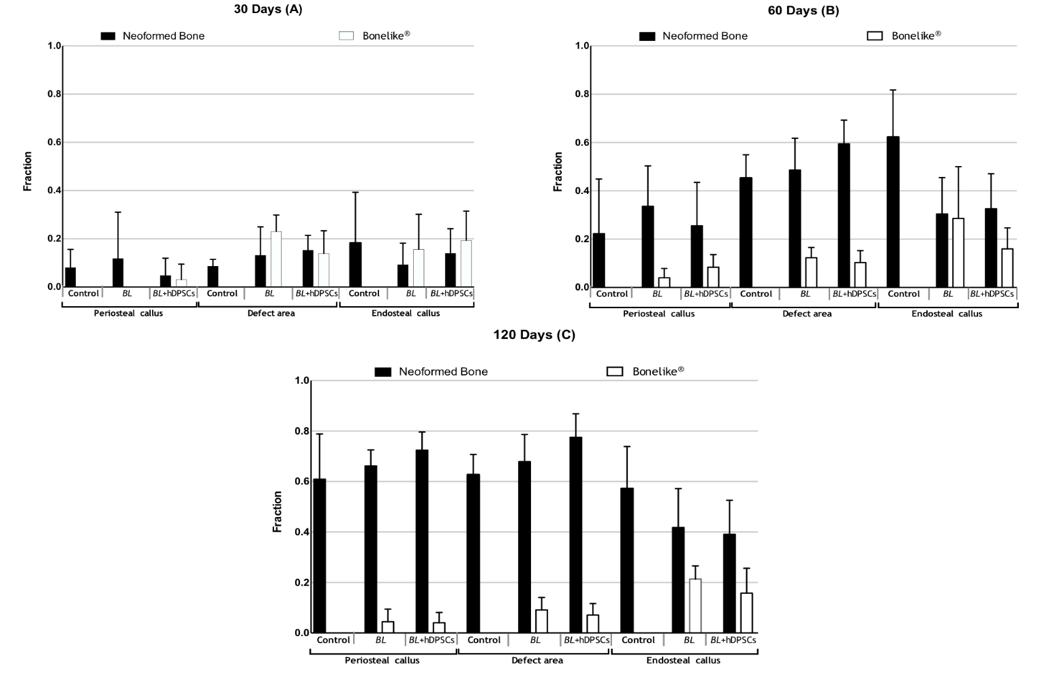

Supplement: Supplementary Data [file rby025_supp.zip › rby025-suppl_data/rby025_Supplementary_Figure_S4.tiff]

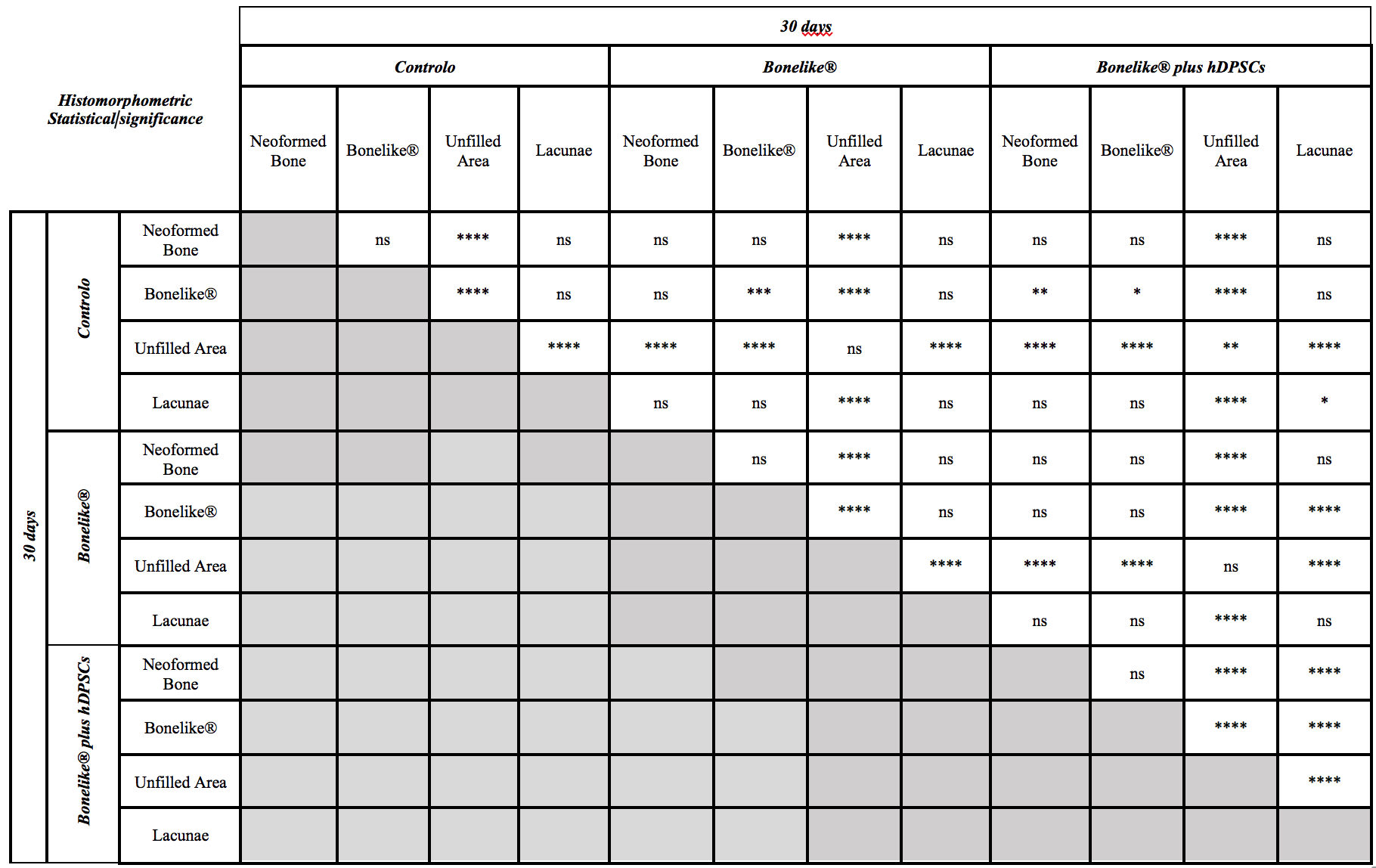

Supplement: Supplementary Data [file rby025_supp.zip › rby025-suppl_data/rby025_Supplementary_Table_S1.tiff]

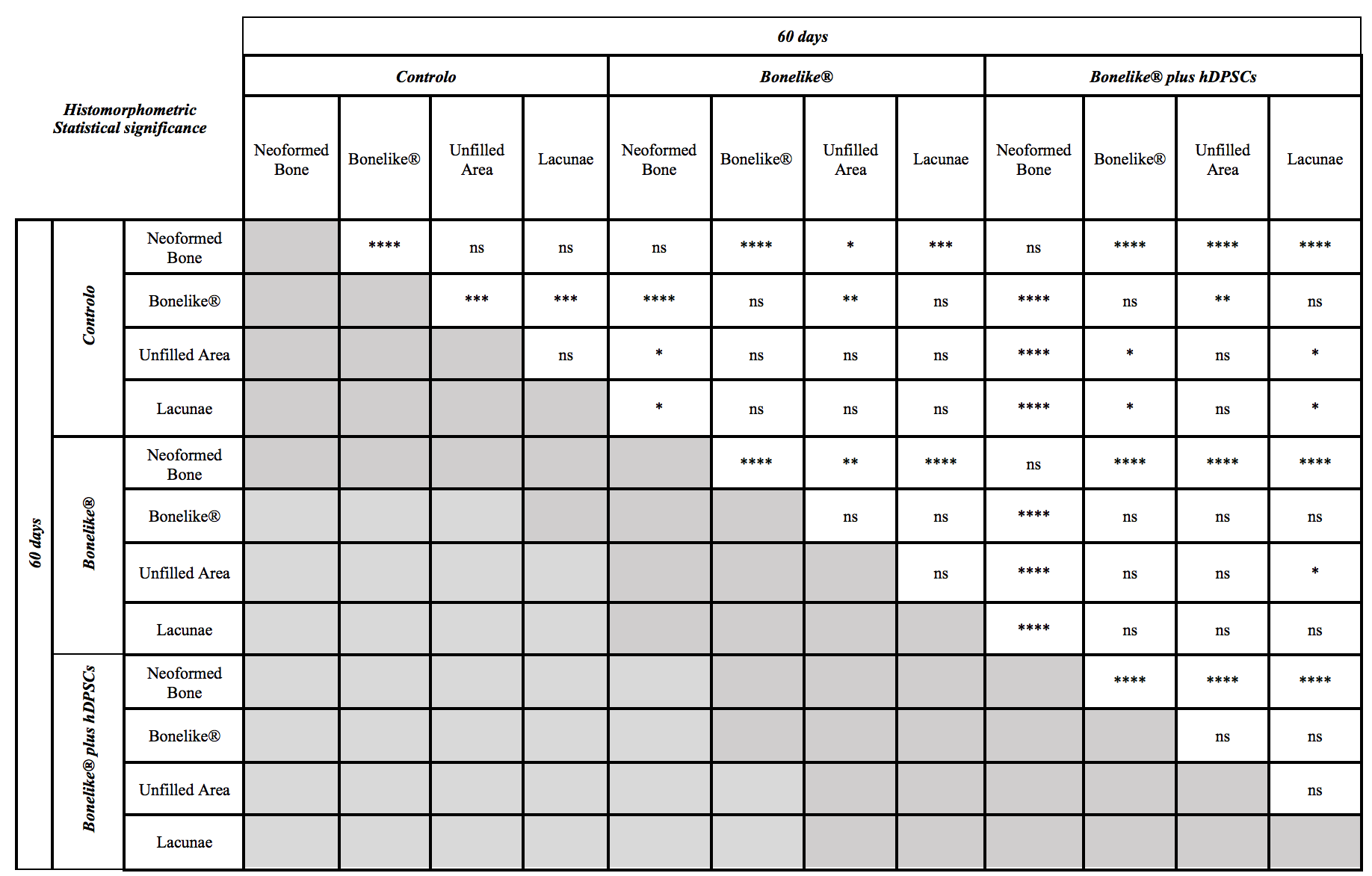

Supplement: Supplementary Data [file rby025_supp.zip › rby025-suppl_data/rby025_Supplementary_Table_S2.tiff]

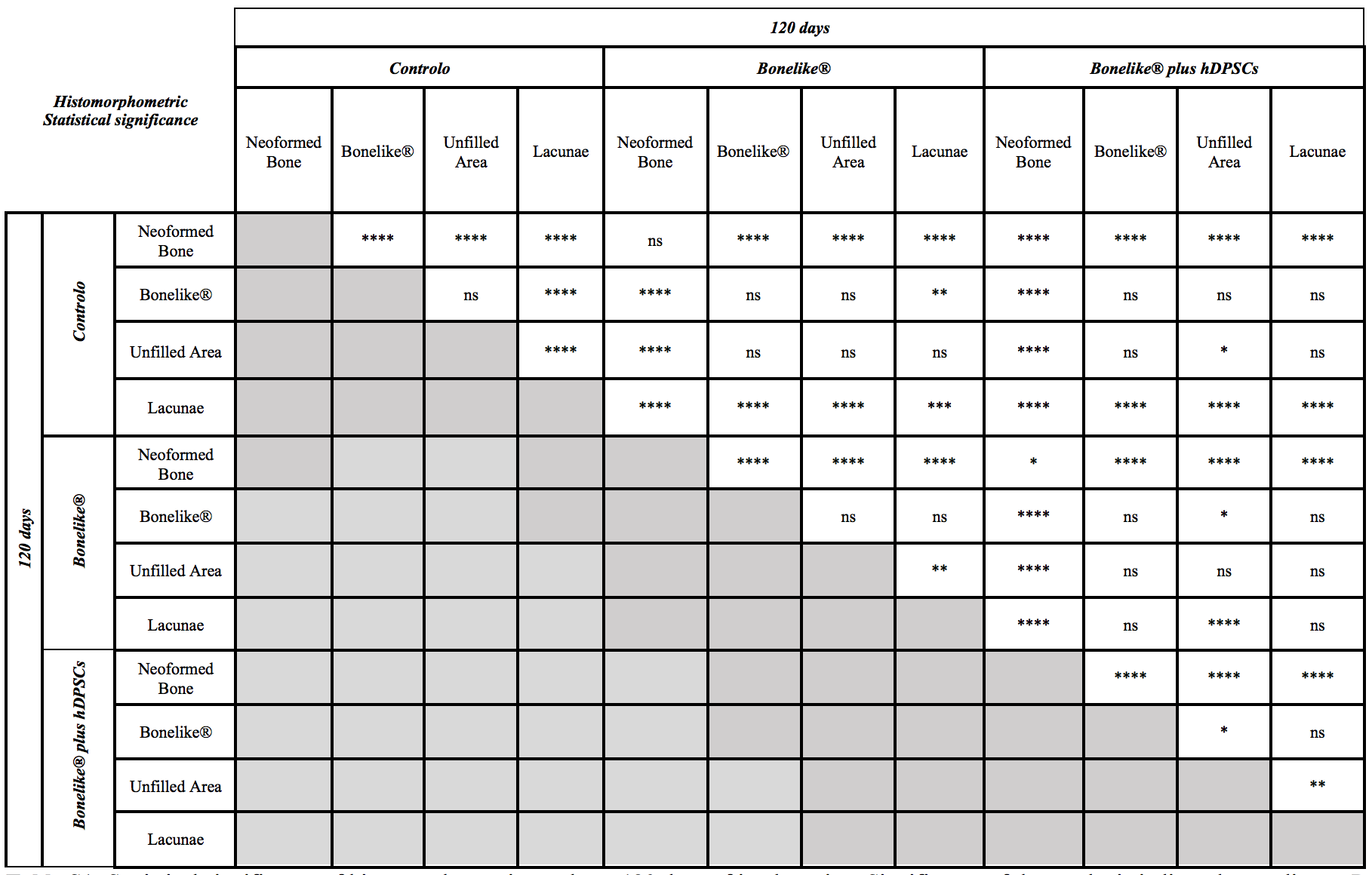

Supplement: Supplementary Data [file rby025_supp.zip › rby025-suppl_data/rby025_Supplementary_Table_S3.tiff]

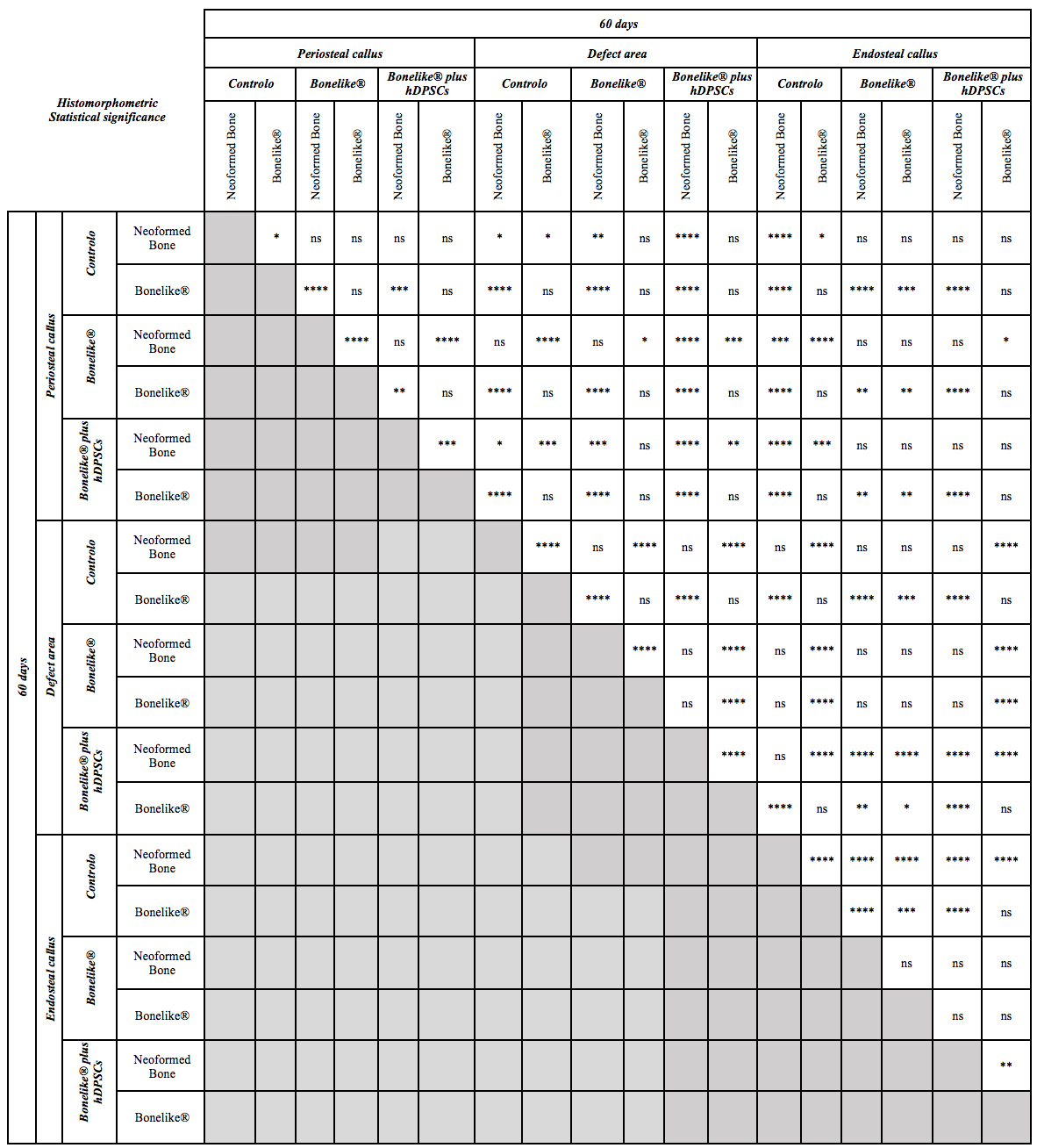

Supplement: Supplementary Data [file rby025_supp.zip › rby025-suppl_data/rby025_Supplementary_Table_S5.tiff]

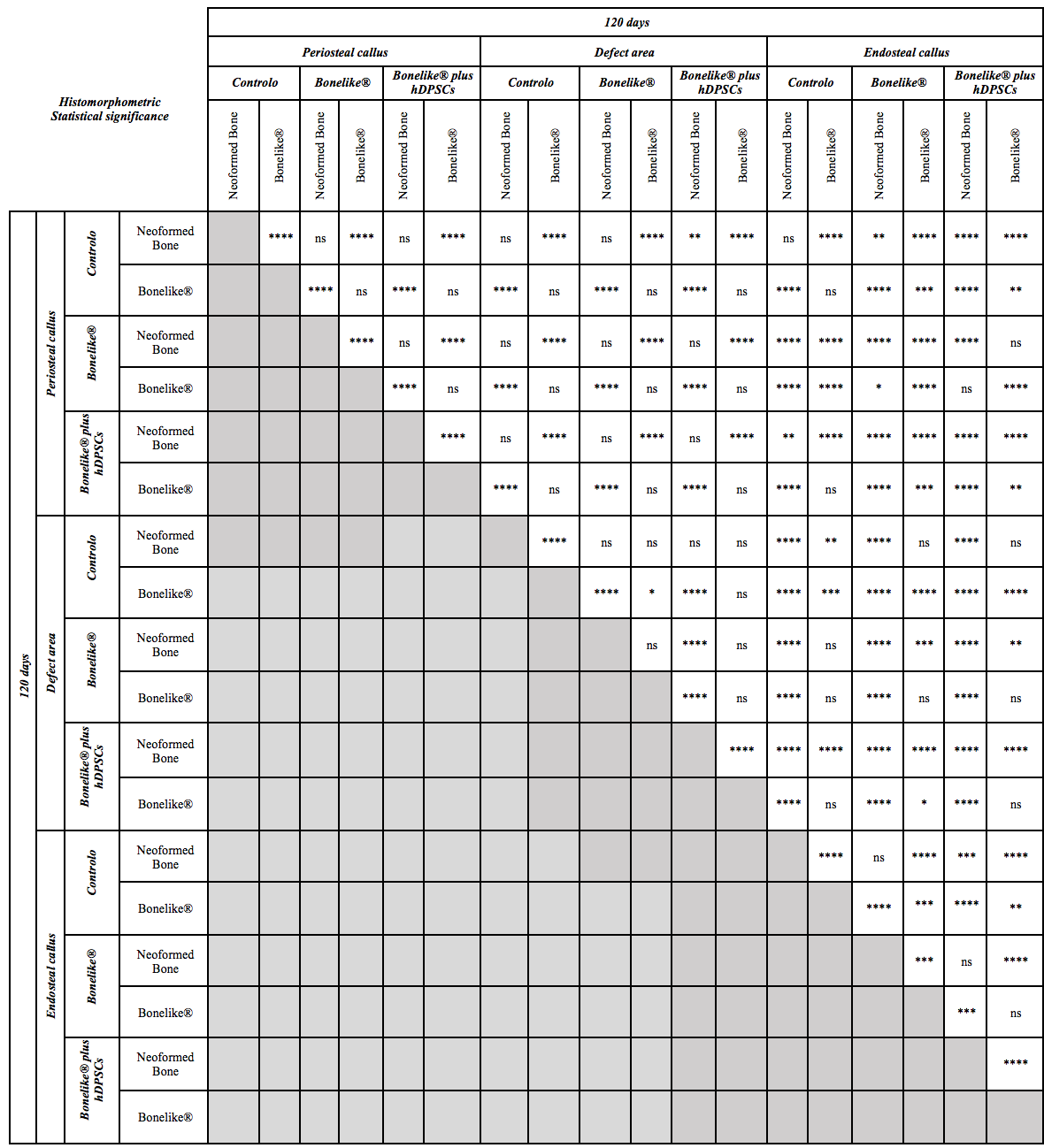

Supplement: Supplementary Data [file rby025_supp.zip › rby025-suppl_data/rby025_Supplementary_Table_S6.tiff]

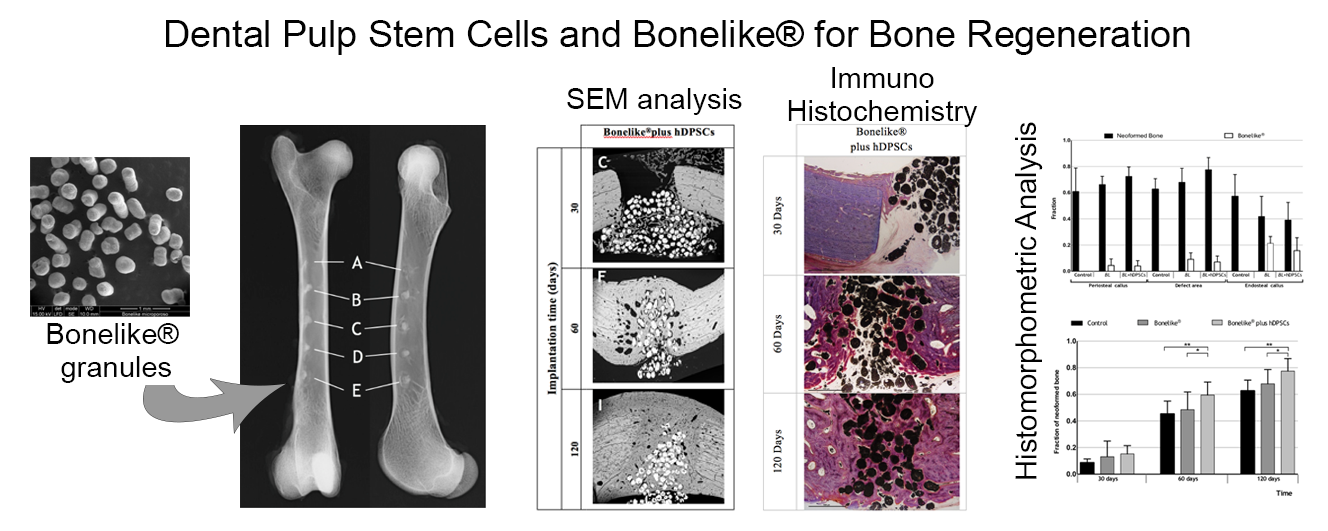

Supplement: Supplementary Data [file rby025_supp.zip › rby025-suppl_data/rby025_Supplementary_GA.tif]

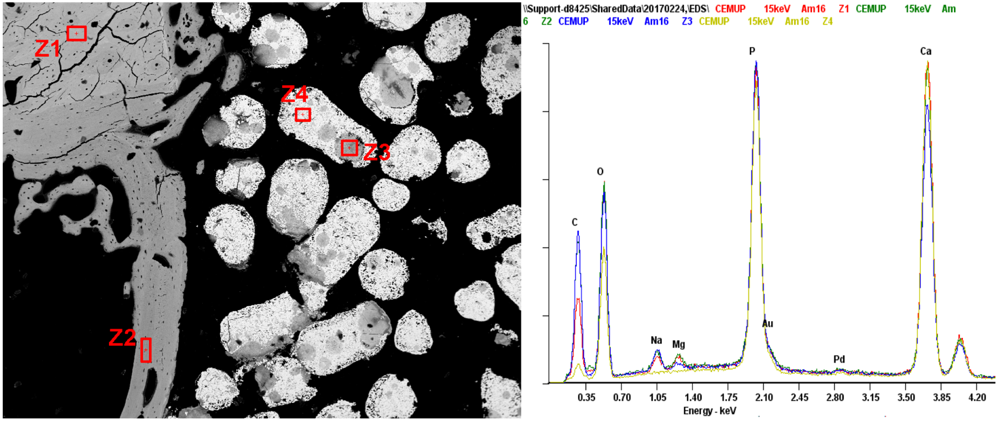

Supplement: Supplementary Data [file rby025_supp.zip › rby025-suppl_data/rby025_Supplementary_Figure_S2.tiff]

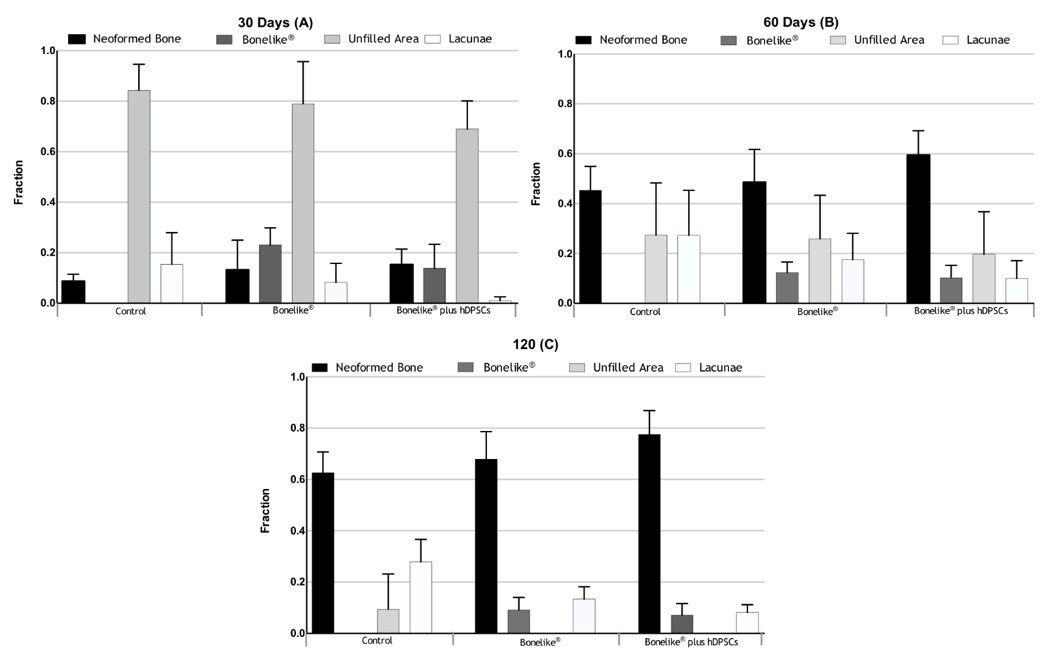

Supplement: Supplementary Data [file rby025_supp.zip › rby025-suppl_data/rby025_Supplementary_Figure_S3.tiff]

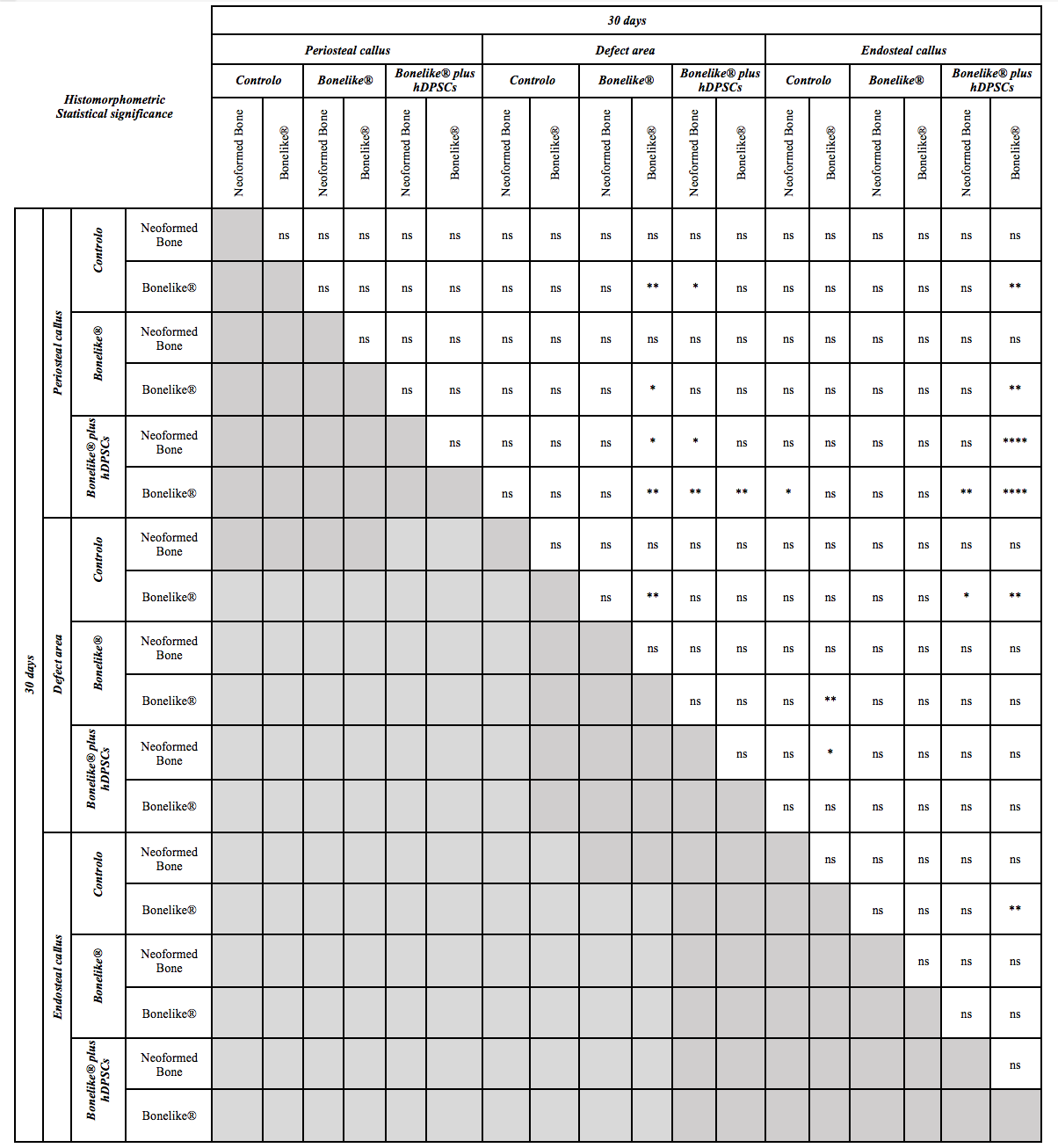

Supplement: Supplementary Data [file rby025_supp.zip › rby025-suppl_data/rby025_Supplementary_Table_S4.tiff]
