# Supplementary material for: Dental pulp stem cells and Bonelike® for bone regeneration in ovine model
Source: Regen Biomater. 2018 Dec 22;6(1):49–59. doi: 10.1093/rb/rby025 (PMC6362823; doi:10.1093/rb/rby025)
Supplement: Supplementary Data [file rby025_supp.zip › rby025-suppl_data/Cover letter Campos Sousa Caseiro et al 2018 reviewrs.docx]

LARGO PROF. ABEL SALAZAR, 2. 4099-003 PORTO

TELEFONE + 351 22 206 22 00

FAX + 351 22 206 22 32

Porto, 01 of October 2018

Dear Editors

*Regenerative Biomaterials*

*Nicholas Peppas and Xingdong Zhang*

**Subject:** Re-submission of the full paper entitled *Dental Pulp Stem Cells and Bonelik^e®^ for Bone Regeneration in Ovine Model* - RB-2018-025.

Enclosed you will find the paper entitled “*Dental Pulp Stem Cells and Bonelike^®^ for Bone Regeneration in Ovine Model.*” by JM Campos, AC Sousa, AR Caseiro, SS Pedrosa, PO Pinto, MV Branquinho, I Amorim, JD Santos, T Pereira, CM Mendonça, A Afonso, LM Atayde, AC Maurício, to be submitted for publication in *Regenerative Biomaterials*, after revision by the authors and according to the reviewers comments.

*“Reviewer: 1*

*Comments to the Author*

*The authors propose a synthetic bone graft (Bonelike^®^) in combination with a cellular system as an alternative for the regeneration of non-critical defects. The preparation, characterization, animal studies and results are very well discussed. My only problem is that the results are presented in a very condensed way without much discussions. Thus, this looks like a medical report, not a paper. I HIGHLY RECOMMEND that the paper be rewritten with more explanations, details in procedures and additional discussion”*

The authors reformulated the results and discussion sections, according to the reviewer recommendations, including a more detailed description of the results and a more interesting discussion. The authors would also like to include two more co-authors to the manuscript - *I Amorim* and *A Afonso* - who actively participated in the histological and histomorphometric analysis and in the revision of the manuscript. The revised paper shows the revised / re-written parts highlighted in yellow.

Thank you in advance for your attention.

Best regards,

On behalf of all authors,

Prof. Ana Colette Maurício (corresponding author)

Departamento de Clínicas Veterinárias

Instituto de Ciências Biomédicas Abel Salazar (ICBAS)

Rua de Jorge Viterbo Ferreira, nº 228

4050-313 Porto, Portugal.

Mobile: +351919071286 ; Phone: +351220428000

Email: ana.colette@hotmail.com, [acmauricio@icbas.up.pt](mailto:acmauricio@icbas.up.pt)
