# Supplementary material for: Dental pulp stem cells and Bonelike® for bone regeneration in ovine model
Source: Regen Biomater. 2018 Dec 22;6(1):49–59. doi: 10.1093/rb/rby025 (PMC6362823; doi:10.1093/rb/rby025)
Supplement: Supplementary Data [file rby025_supp.zip › rby025-suppl_data/Cover letter Campos Sousa Caseiro et al 2018.docx]

LARGO PROF. ABEL SALAZAR, 2. 4099-003 PORTO

TELEFONE + 351 22 206 22 00

FAX + 351 22 206 22 32

Porto, 23 of January 2018

Dear Editors

*Regenerative Biomaterials*

*Nicholas Peppas*

*Xingdong Zhang*

**Subject:** Submission of the full paper entittled *Dental Pulp Stem Cells and Bonelike® for Bone Regeneration in Ovine Model.*

Enclosed you will find the paper entitled “*Dental Pulp Stem Cells and Bonelike® for Bone Regeneration in Ovine Model.*” by JM Campos, AC Sousa, AR Caseiro, SS Pedrosa, PO Pinto, MV Branquinho, JD Santos, T Pereira, CM Mendonça, LM Atayde, AC Maurício, to be submitted for publication in *Regenerative Biomaterials.*

The sought for effective new cellular therapeutic strategies for improving tissue regeneration represents one of the hot topics in biomedicine. The main aim of this study has been to evaluate the capability of human dental pulp stem cells (hDPSCs) associated to a well-characterized synthetic substitute – Bonelike^®^ - to promote bone regeneration, using an animal model very close to humans, the ovine. Because of the growing interest in mesenchymal stem cells employment in Regenerative Medicine and Tissue Engineering emerging areas associated to bioactive biomaterials, we think that the results of our paper can be interesting for a interdisciplinary readership working in nerve regeneration and thus we decided to submit it to *Regenerative Biomaterials.*

This work is original in that it has not been published before or submitted for publication elsewhere, and will not be submitted elsewhere before a decision has been taken as to its acceptability in this Journal where you are Editor. Each author meets the criteria for authorship and assumes the corresponding responsibility.

Thank you in advance for your attention.

Best regards,

On behalf of all authors,

Prof. Ana Colette Maurício (corresponding author)

Departamento de Clínicas Veterinárias

Instituto de Ciências Biomédicas Abel Salazar (ICBAS)

Rua de Jorge Viterbo Ferreira, nº 228

4050-313 Porto, Portugal.

Mobile: +351919071286 ; Phone: +351220428000

Email: ana.colette@hotmail.com, [acmauricio@icbas.up.pt](mailto:acmauricio@icbas.up.pt)
